# Phenotypic and genotypic features of the *Mycobacterium tuberculosis* lineage 1 subgroup in central Vietnam

**DOI:** 10.1038/s41598-021-92984-5

**Published:** 2021-06-30

**Authors:** Nguyen Thi Le Hang, Minako Hijikata, Shinji Maeda, Akiko Miyabayashi, Keiko Wakabayashi, Shintaro Seto, Nguyen Thi Kieu Diem, Nguyen Thi Thanh Yen, Le Van Duc, Pham Huu Thuong, Hoang Van Huan, Nguyen Phuong Hoang, Satoshi Mitarai, Naoto Keicho, Seiya Kato

**Affiliations:** 1 NCGM-BMH Medical Collaboration Center, Hanoi, Vietnam; 2 Department of Pathophysiology and Host Defense, The Research Institute of Tuberculosis, JATA, Tokyo, Japan; 3 Faculty of Pharmaceutical Sciences, Hokkaido University of Science, Hokkaido, Japan; 4 Department of Microbiology, Da Nang Lung Hospital, Da Nang, Vietnam; 5 Da Nang General Hospital, Da Nang, Vietnam; 6 Hanoi Lung Hospital, Hanoi, Vietnam; 7 Department of Microbiology, Hanoi Lung Hospital, Hanoi, Vietnam; 8 Department of Mycobacterium Reference and Research, The Research Institute of Tuberculosis, JATA, Tokyo, Japan; 9 The Research Institute of Tuberculosis, JATA, Japan Anti-Tuberculosis Association, 3-1-24 Matsuyama, Kiyose, Tokyo 204-8533 Japan; 10 National Center for Global Health and Medicine, Tokyo, Japan

**Keywords:** Tuberculosis, Genetic variation

## Abstract

*Mycobacterium tuberculosis* (Mtb) has different features depending on different geographic areas. We collected Mtb strains from patients with smear-positive pulmonary tuberculosis in Da Nang, central Vietnam. Using a whole genome sequencing platform, including genome assembly complemented by long-read-sequencing data, genomic characteristics were studied. Of 181 Mtb isolates, predominant Vietnamese EAI4_VNM and EAI4-like spoligotypes (31.5%), ZERO strains (5.0%), and part of EAI5 (11.1%) were included in a lineage-1 (L1) sublineage, i.e., L1.1.1.1. These strains were found less often in younger people, and they genetically clustered less frequently than other modern strains. Patients infected with ZERO strains demonstrated less lung infiltration. A region in RD2bcg spanning six loci, i.e., *PE_PGRS35*, *cfp21*, Rv1985c, Rv1986, Rv1987, and *erm(37)*, was deleted in EAI4_VNM, EAI4-like, and ZERO strains, whereas another 118 bp deletion in *furA* was specific only to ZERO strains. L1.1.1.1-sublineage-specific deletions in *PE_PGRS4* and *PE_PGRS22* were also identified. RD900, seen in ancestral lineages, was present in majority of the L1 members. All strains without IS*6110* (5.0%) had the ZERO spoligo-pattern. Distinctive features of the ancestral L1 strains provide a basis for investigation of the modern versus ancestral Mtb lineages and allow consideration of countermeasures against this heterogeneous pathogen.

## Introduction

Analyses of global strains of *Mycobacterium tuberculosis* (Mtb) have revealed major lineages, referred to as L1 to L7^1,2^. Recently, L8 and L9 have been added to the original seven^3,4^. In response, Napier et al.^5^ updated the original single nucleotide polymorphism (SNP) barcoding system^6^, using more than 30,000 Mtb strains, to classify their (sub)lineages more systematically.

Mtb genotypes vary from population to population and are highly geographically structured^7–9^. Different Mtb lineages tend to present with different characteristics and virulence phenotypes, including host immune response regulation, transmissibility, disease severity^10^, drug-resistance profiles^11^, and efficacy to vaccination. Beijing genotype strains in L2 have been shown to have an association with drug resistance, treatment failure, early recurrence^10,12,13^, and increased risk of transmission chains globally, whereas L1 strains confer local risk in parts of Asia and Africa^8,9^. A better understanding of phenotypic variations caused by genetic diversity of Mtb strains is important when attempting to improve TB control measures.

Vietnam, located in southeast Asia, is one of 30 countries with a high TB burden. Extending nearly 2000 km from the north through the central area to the south, different distributions of Mtb lineages in each of these areas of Vietnam have been found. Beijing genotype strains, a major sublineage of L2, are predominant in the northern and southern regions, whereas EAI strains in L1 are more frequently seen in the central area^14–16^. The EAI4_VNM spoligotype, which is characterized by the absence of spacers 26 and 27, 29 to 32, and 34 of 43 in the clustered regularly interspaced short palindromic repeat (CRISPR) locus of the Mtb genome^17^, belongs to a major L1 subgroup in Vietnam^18^. In our previous study^16^ in Da Nang city, a central area of Vietnam, we showed that these strains form a clade that is discrete from other EAI strains in different geographical regions in Asia and Africa. Carrying only a few IS*6110* copies is also a characteristic of these Vietnamese strains^16,19^. For a comprehensive understanding of the genotypic and phenotypic characteristics of these strains, we used whole genome sequencing (WGS) to analyze the whole sample set of 181 Mtb strains with clinico-epidemiological data in Da Nang in central Vietnam. We also compared the results with a northern study cohort, another southern cohort studied by others, and reports from other countries.

## Results

### Distribution of Mtb lineages/sublineages in Da Nang city

We recruited 251 patients with smear-positive active pulmonary TB who directly visited Da Nang Lung Hospital or who were referred via district TB centers from January 2015 to November 2016. The median patient age was 44.5 years old (interquartile range IQR 33.0–53.9), 81.3% were males, 85.3% were new cases, and 14.7% had TB treatment history. WGS (Illumina) was successful in Mtb isolates from 181 of the 251 patients; L1 accounted for 48.1% (87/181), L2 for 35.3% (64/181), and L4 for 16.6% (30/181). By spoligotyping, EAI accounted for 42.5% (77/181), which comprised an EAI4_VNM subgroup (57/181 isolates or 31.5%, of which 8/57 were EAI4_VNM-like) and an EAI5 subgroup (20/181 or 11.1%, of which 6/20 were EAI5-like, following the criterion described in the Methods and Materials section). The spoligo international type (SIT) numbers are also presented in Table 1. A group showing a SIT405 or SIT802 spoligotype pattern (hereafter called “ZERO” types following the SITVITWEB database, also known as “zero-copy” following SITVIT2) accounted for 5.0% (9/181). Modern Beijing strains accounted for 24.9% (45/181) and ancient Beijing strains accounted for 8.8% (16/181; Table 1).

**Table 1:** Proportions of Mtb lineages/sublineages by the SNP barcode and clades by *in-silico* spoligotyping in Da Nang samples (N = 181).

(Sub)Lineage/spoligotype	The spoligo international type (SIT) numbers	Freq. (n)	Proportion per lineage (%)	Overall proportion (%)
**Lineage 1 (n = 87)**				**48.1**
L1.1.1		9	10.3	5.0
EAI5	234, 236, 792	6	6.9	3.3
EAI5-like	NA	3	3.4	1.7
L1.1.1.1		78	89.7	43.1
EAI4_VNM	139, 456, 564, 514, 622, 1731, 2722, Orphan	49	56.3	27.1
EAI4_VNM-like	3196, NA	8	9.2	4.4
ZERO	405, 802	9	10.3	5.0
EAI5	236, 458, 618	8	9.2	4.4
EAI5-like	NA	3	3.4	1.7
Unknown	NA	1	1.1	0.6
**Lineage 2 (n = 64)**				**35.3**
L2.1 (proto Beijing)	623, NA	3	4.7	1.7
L2.2.2 (ancient Beijing)	1, 269	2	3.1	1.1
L2.2.1 (ancient Beijing)	1, 190	14	21.9	7.7
L2.2.1 (modern Beijing)	1, 190	38	59.4	21.0
L2.2.1.1 (modern Beijing)	1	7	10.9	3.9
**Lineage 4 (n = 30)**				**16.6**
L4.1 (Unknown, X1)	336, NA	8	26.7	4.4
L4.1.1 (X1)	119	2	6.7	1.1
L4.1.2 (T)	73	1	3.3	0.6
L4.1.2.1 (H2, T2)	2, 888, 52	3	10.1	1.7
L4.2.2. (T1)	51	4	13.3	2.2
L4.3.1 (LAM9)	42	1	3.3	0.6
L4.3.3 (LAM9)	388	1	3.3	0.6
L4.4.1.2 (Unknown)	NA	3	10.0	1.7
L4.4.2 (T2, Unknown)	52, NA	2	6.7	1.1
L4.5 (H3)	50	1	3.3	0.6
L4.8 (T1, Unknown)	53, NA	4	13.3	2.2

*Mtb* *Mycobacterium tuberculosis*, *NA* not available, *SNP* single nucleotide polymorphism.

Updated SNP barcode by Napier et al.^5^.

### Distribution of L1 subgroups in the phylogenetic tree

When L1 strains in Da Nang were assessed by the updated SNP barcoding system^5^, L1.1.1.1 and L1.1.1 accounted for 89.7% (78/87) and 10.3% (9/87), respectively. EAI4_VNM, EAI4_VNM-like, and ZERO strains were included in L1.1.1.1 (66/78 or 84.6%), whereas EAI5 and EAI5-like strains partially contributed to the L1.1.1.1 subgroup (11/78 or 14.1%) and L1.1.1 subgroup (9/9 or 100.0%; Table 1).

Among the 332 strains obtained in our previous study from Hanoi in northern Vietnam ^15,20^, all EAI4_VNM and EAI4_VNM-like strains also belonged to L1.1.1.1 (78.9% [60/76]), whereas EAI5 and EAI5-like strains contributed to both L1.1.1 (5/6 [83.3%]) and L1.1.1.1 (10/76 [13.2%]; table not shown). There were no ZERO strains in this Hanoi cohort. In another data set from Ho Chi Minh city in southern Vietnam^21^, the distribution of major lineages/sublineages was closer to that of northern Vietnam rather than central Vietnam; 60.2% were Beijing strains, 13.0% were EAI4_VNM strains and 3.8% were EAI5 strains. ZERO strains accounted for 1.7% (27/1,635) of this cohort (Table not shown).

**Figure 1:**
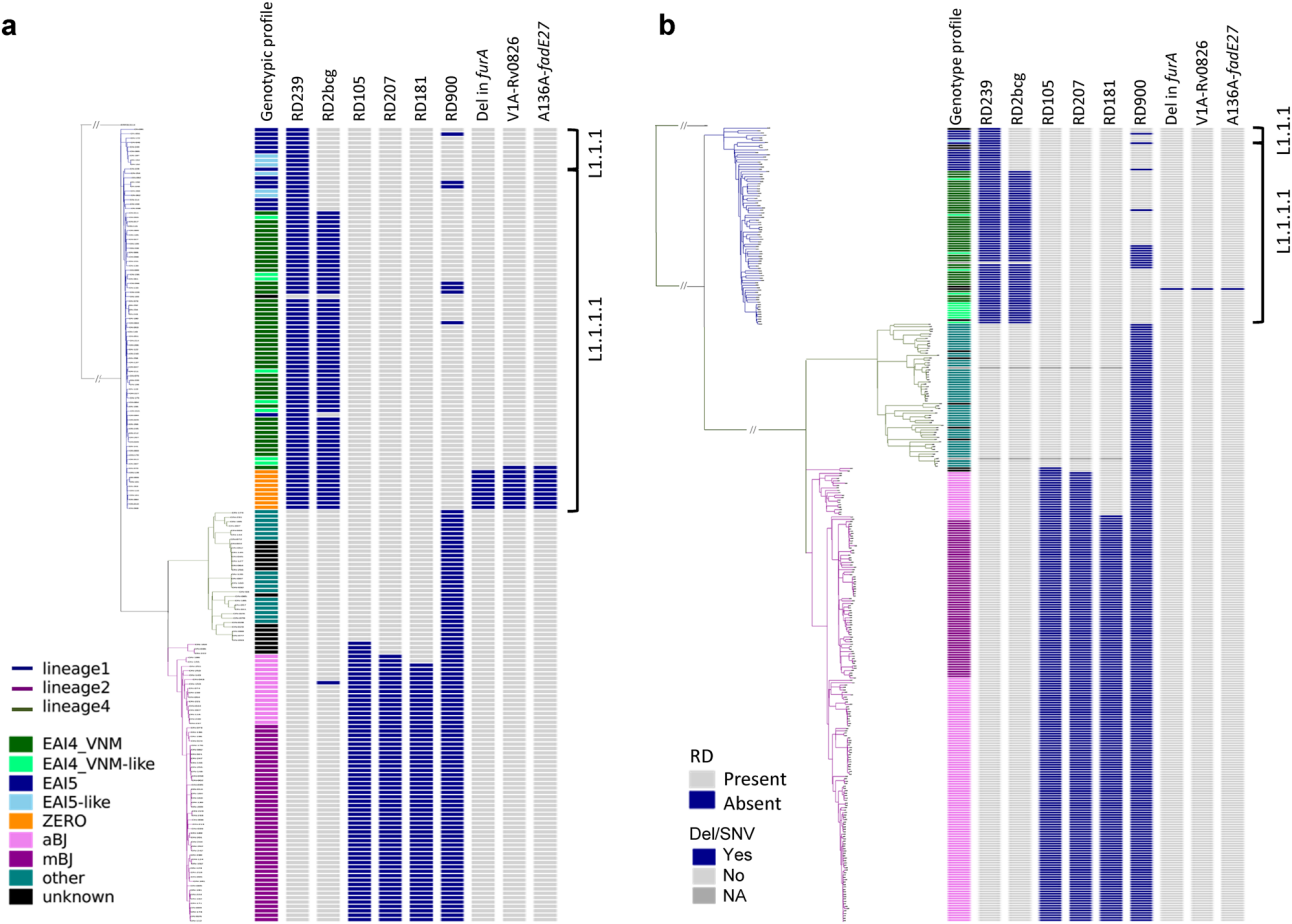
Phylogenetic tree of 181 Da Nang strains (**a**) and 332 Hanoi strains (the northern Vietnam data set) (**b**), constructed using variants after mapping with H37Rv. Phylogenetic trees were constructed with the maximum likelihood method using RAxML version 8.2.8 (https://github.com/stamatak/standard-RAxML) and visualized with plotTree for python v2.7 (https://github.com/katholt/plotTree). Regions of difference (RDs), deletions, and SNVs in correlation with Mtb clades are depicted. *Mtb* *Mycobacterium tuberculosis*, *SNV* single nucleotide variant, *Del* deletion, *aBJ* ancient Beijing, *mBJ* modern Beijing, *NA* not available.

**Figure 2:**
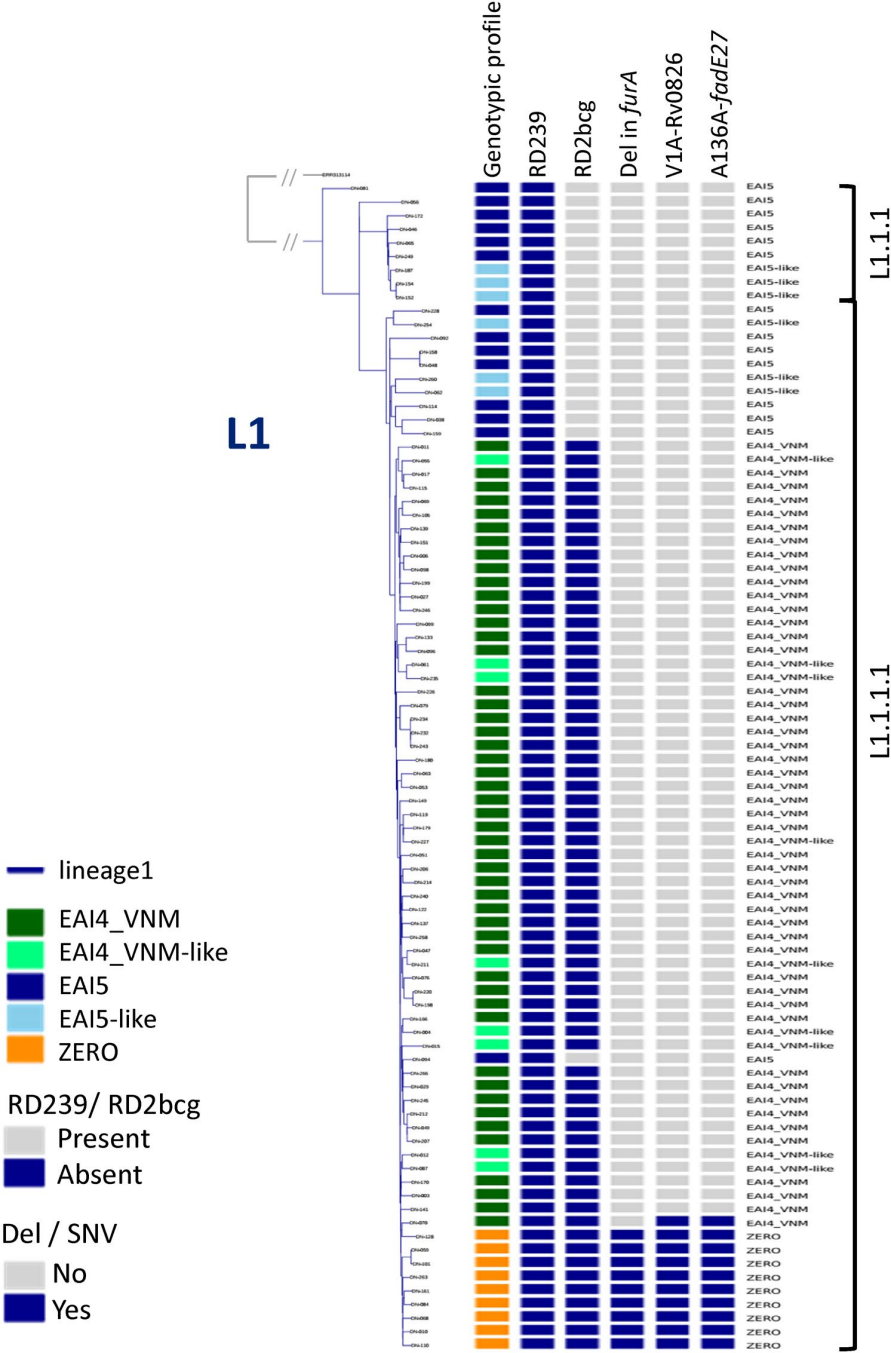
Phylogenetic tree of 87 lineage-1 strains from the Da Nang cohort. AP018033.1 (EAI4_VNM) was used as a reference genome. Phylogenetic trees were constructed with the maximum likelihood method using RAxML version 8.2.8 (https://github.com/stamatak/standard-RAxML) and visualized with plotTree for python v2.7 (https://github.com/katholt/plotTree). RD239, RD2bcg, deletion in *furA,* and SNVs in correlation with Mtb clades are shown. *Mtb* *Mycobacterium tuberculosis*, *SNV* single nucleotide variant, *Del* deletion.

The L1 branches consisted of two distinct subgroups that were close to each other, that is, L1.1.1 and L1.1.1.1 (Fig. 1a). EAI4_VNM- and EAI4_VNM-like strains were distributed closely together inside the L1.1.1.1 branches. EAI5 and EAI5-like strains are located in L1.1.1 or L1.1.1.1. ZERO spoligotype strains were included in L1.1.1.1. These patterns were similar to those of northern Vietnam (Fig. 1b), even when the reference genome was changed to AP018033.1 (EAI4_VNM, L1; Fig. 2).

Because EAI4_VNM, EAI4_VNM-like, and ZERO strains were genetically close to each other and contributed to the majority of L1.1.1.1, we further characterized L1.1.1.1 genotypically and phenotypically, as compared with other strains in our cohort.

### Demographic findings

In our study, the median age of TB onset was 44.5 years (yo; IQR [31.7–54.8]), which was slightly higher than that of the Hanoi cohort (39.4 [29.7–50.5]; *P* = 0.0318 by Wilcoxon rank-sum test; table not shown). When age at onset was younger, L1.1.1.1 was less frequently observed in the patients (30.2%, 46.5%, and 52.4% in the strata of ages < 35, 35.0–54.9, and ≥ 55.0, respectively; *P* = 0.0255 by the Cochran–Armitage trend test; table not shown). A similar trend was also observed in the Hanoi cohort (17.9%, 21.6%, and 40.0%, respectively, *P* = 0.0045; table not shown). The opposite trend of L2/Beijing strains after excluding the L2.1 proto-Beijing genotype did not reach significance in our study (41.5%, 34.9%, and 28.6%, respectively; *P* = 0.1882), whereas higher frequencies in younger patients were observed significantly in Hanoi (61.2%, 60.8%, and 36.0%, respectively; *P* = 0.0116, table not shown).

Using logistic regression models, we also found that age was significantly associated with L1.1.1.1 before (odds ratio [OR] with 95% confidence interval [CI] = 1.60 [1.06–2.44]) and after (adjusted odds ratio [aOR] = 1.56 [95% CI 1.01–2.42]) adjustment for the patient’s gender and body mass index (BMI; Table 2a). Other demographic factors, such as BMI, educational level, occupation, history of TB treatment, or living area, were not associated with the L1.1.1.1 subgroup (table not shown).

**Table 2:** Univariate and multivariate analyses using logistic regression models.

Factors	n/N (%)	Univariate		Multivariate	
		OR	95% CI	aOR**	95% CI
**(a)** Mtb L1.1.1.1 sublineage associated with age strata before and after adjustment for other factors					
Age strata* (increased by one level)	NA	1.60	1.06–2.44	1.56	1.01–2.42
Gender					
Male	67/150 (44.7)	Ref		Ref	
Female	11/31 (35.5)	0.68	0.31–1.52	0.85	0.36–1.99
BMI (increased by one unit)	NA	1.03	0.91–1.17	1.03	0.91–1.17
**(b)** Drug resistance*** associated with lineage/sublineage before and after adjustment for other factors					
Lineage/sublineage					
Other	7/38 (18.4)	Ref		Ref	
L1.1.1.1	15/72 (20.8)	1.17	0.43–3.16	1.33	0.48–3.69
L2/Beijing****	24/56 (42.9)	3.32	1.25–8.82	3.57	1.30–9.77
Age strata (increased by one level)	NA	0.66	0.40–1.07	0.62	0.36–1.07
Gender: Male	39/139 (28.1)	Ref		Ref	
Female	7/27 (25.9)	0.90	0.35–2.29	0.58	0.21–1.66
BMI (increased by one unit)	NA	0.94	0.81–1.09	0.96	0.82–1.13
**(c)** Genotypic clusters (≤ 12 pairwise SNV difference) associated with lineage/sublineage before and after adjustment for other factors					
Lineage/sublineage					
Other	16/42 (38.1)	Ref		Ref	
L1.1.1.1	5/78 (6.4)	0.11	0.04–0.33	0.11	0.03–0.32
L2/Beijing****	6/61 (9.8)	0.18	0.06–0.51	0.16	0.06–0.48
Age strata (increased by one level)	NA	0.89	0.51–1.58	1.15	0.61–2.15
Gender: Male	21/150 (14.0)	Ref		Ref	
Female	6/31 (19.4)	1.47	0.54–4.02	1.85	0.59–5.82
BMI (increased by one unit)	NA	1.02	0.86–1.20	1.00	0.82–1.21

*BMI* body mass index, *NA* not applicable, *Mtb* *Mycobacterium tuberculosis*, *SNV* single nucleotide variant.

*Age strata: < 35.0, 35.0–54.9, ≥ 55 years old.

**aOR: adjusted odds ratio.

***Phenotypic resistance to at least one of the four major drugs (Rifampicin, Isoniazid, Ethambutol, and Streptomycin).

****Except L2.1, the proto-Beijing genotype.

### Drug-resistance profiles

Of the 173 patients in which phenotypic drug susceptibility test results were available, resistance to any of the four major drugs (Isoniazid [INH], Rifampicin, Ethambutol, and Streptomycin) was observed in 28.3% (49/173). L2/Beijing strains were positively associated with drug resistance before (OR = 3.32 [95% CI 1.25–8.82]) and after (aOR = 3.57 [95% CI 1.30–9.77]) adjustment for age, gender, and BMI in logistic regression models, whereas L1.1.1.1 did not demonstrate any significant association with drug resistance before (OR = 1.17 [0.43–3.16]) and after (aOR = 1.33 [0.48–3.69]) adjustment (Table 2b). Drug resistance was also not different between the ZERO and nonZERO strains within the L1 subgroup (22.2% vs. 17.0%; *P* = 0.655 by Chi-squared test).

Gene variants that confer resistance to 21 drugs were evaluated by the TBProfiler’s known mutation list^5^. Among them, *katG*-S315T was the most frequent single mutation against INH and was carried by 24 (13.3%) of 181 isolates. Mutations in *rpoB* were observed in 7 (3.9%), in *embB* in 9 (5.0%), and in *rpsL* in 24 (13.3%) isolates (Table not shown). Similar to phenotypic drug resistance, L2/Beijing strains were associated with at least one of the above mutations, either in univariate or in multivariate analysis (OR = 3.47 [95% CI 1.33–9.06] and aOR = 3.78 [1.42–10.09], respectively), and L1.1.1.1 was not (OR = 1.19 [0.44–3.20] and aOR = 1.29 [0.47–3.53], respectively; table not shown).

### Genotypic clusters in the L1.1.1.1 subgroup

When no more than 12 single nucleotide variants (SNVs) was set as the threshold for genotypic clusters, the proportion of clustered strains was 14.9% (27/181) in the entire Da Nang cohort; those of the L1.1.1.1 and L2/Beijing sublineages were 6.4% (5/78) and 9.8% (6/61), respectively, and were lower than others, mainly L4 strains, by both univariate analyses (OR = 0.11 [95% CI 0.04–0.33] and 0.18 [0.06–0.51], respectively) and multivariate analyses (aOR = 0.11 [95% CI 0.03–0.32] and 0.16 [0.06–0.48], respectively) using logistic regression models in our study cohort (Table 2c). In the Hanoi cohort, the L2/Beijing genotype was positively associated with clustered strains (OR = 3.77 [95% CI 1.98–7.15], aOR = 3.82 [2.00–7.28]), whereas L1.1.1.1 was negatively associated (OR = 0.30 [0.11–0.83], aOR = 0.32 [0.11–0.89]) (Table not shown).

Additional characteristics of ZERO strains are shown in Table 3a. Two of nine strains, which harbored the *katG*-S315T mutation, were mono-resistant to INH phenotypically. The number of lung zones with infiltrates shown on a chest X-ray was low (median = 1 [0–1]), and it was lower compared with that in patients infected with other strains (2 [1–4], *P* = 0.013 by a Wilcoxon rank-sum test; table not shown). Spreading of the infiltrate was reversely associated with ZERO strains before (OR = 0.41 [95% CI 0.17–0.98]) and after (aOR = 0.33 [0.12–0.90]) adjustment for the patient’s age, culture, and smear grade before treatment, presence of cavities on chest X-ray, or Mtb harboring the *katG*-S315T mutation (Table 3b). All ZERO strains were not genotypically clustered (Table 3a).

**Table 3:** Characteristics of ZERO strains (a) and multivariate analysis using logistic regression models to investigate factors possibly associated with ZERO strains (b).

(a)	Patients ID								
Characteristics	DN-010	DN-059	DN-068	DN-084	DN-101	DN-110	DN-128	DN-161	DN-263
Gender, age	M, 48.6	M, 50.8	M, 26.9	M, 60.1	M, 44.5	M, 61.2	M, 42.1	M, 28.7	M, 64.6
Body mass index	18.7	17.2	19.5	20.2	14.6	17.9	20.0	20.8	15.2
Type of cases	New	New	New	New	New	Retreated	New	New	New
HIV	Neg	Neg	Neg	Neg	Neg	Neg	Neg	Neg	Neg
Phenotypic drug susceptibility									
Isoniazid 0.2 µg/mL	S	S	S	S	S	S	R	R	S
Isoniazid 1 µg/mL	S	S	S	S	S	S	R	S	S
Rifampicin	S	S	S	S	S	S	S	S	S
Streptomycin	S	S	S	S	S	S	S	R	S
Ethambutol	S	S	S	S	S	S	S	S	S
Cavity area* on chest X-ray	NA	1	1	4	0	0	0	0	NA
Infiltrate area* on chest X-ray	NA	1	0	0	1	4	1	1	NA
Smear grade before treatment	2 +	3 +	1 +	1 +	3 +	1 +	2 +	3 +	2 +
Culture grade before treatment	3 +	1 +	2 +	Scanty	2 +	2 +	1 +	2 +	1 +
Sublineage classification	L1.1.1.1	L1.1.1.1	L1.1.1.1	L1.1.1.1	L1.1.1.1	L1.1.1.1	L1.1.1.1	L1.1.1.1	L1.1.1.1
Spoligotype	ZERO	ZERO	ZERO	ZERO	ZERO	ZERO	ZERO	ZERO	ZERO
SIT	405	405	405	405	405	405	802	405	405
RD239	Del	Del	Del	Del	Del	Del	Del	Del	Del
RD2bcg	Del	Del	Del	Del	Del	Del	Del	Del	Del
*katG*-S315T mutation	No	No	No	No	No	No	Yes	Yes	No
Clustered	No	No	No	No	No	No	No	No	No
Copy number of IS*6110*	0	0	0	0	0	0	0	0	0

(b)		Univariate analysis				Multivariate analysis			
Factors		OR		95% CI		Adjusted OR		95% CI	
Age (increased by one year)		1.02		0.97–1.07		1.01		0.94–1.08	
Smear grade before treatment		1.14		0.60–2.19		1.5		0.65–3.48	
Culture grade before treatment		0.84		0.36–1.95		0.69		0.23–2.04	
Cavity area* on chest X-ray		1.74		0.73–4.17		1.45		0.57–3.68	
Infiltrate area* on chest X-ray		0.41		0.17–0.98		0.33		0.12–0.90	
*katG*-S315T mutation									
No		Ref				Ref			
Yes		1.95		0.38–9.98		6.62		0.93–47.12	

*HIV* human immunodeficiency virus, *M* male, *S* sensitive, *R* resistant, *OR* odds ratio, 95% CI: 95% confidence interval.

*Number of lung zones.

### Genetic variants specific to EAI4_VNM and ZERO strains in L1.1.1.1

A recent report demonstrated that the CRISPR locus has variants in direct-repeat and spacer sequences^22^. Similar to their report, only L1.1.1 and L1.1.1.1 (EAI5, EAI5-like, EAI4_VNM, EAI4_VNM-like, and ZERO strains in our study) had DR1 (GTCGTCAGACCCAAAACCCCGAGAGGGGACGGGAAC, an underlined SNV in the direct repeat), whereas esp38(1) (TGCCCCAGCGTTTAGCGATCACAACACCAACTAATG, an underlined SNV in the spacer) was not observed in ZERO strains as they lost a region spanning spacer 38 (SIT405 and 802).

The RD-Analyzer^23^ was used to screen 31 standard regions of difference (RDs). All L1 isolates had the RD239 deletion, whereas all L2 isolates had the RD105 deletion. The Beijing genotype had the RD207 deletion, and most of them had the RD181 deletion, as expected^15^. We further noticed that all isolates in the EAI4_VNM, EAI4_VNM-like, and ZERO strains had a deletion in RD2bcg, whereas this deletion was not observed in L1.1.1 or L1.1.1.1 EAI5 strains (Fig. 1). The deletion spanning six genetic loci; i.e., *PE_PGRS35* (Rv1983), *cfp21* (Rv1984c), Rv1985c, Rv1986, Rv1987, and *erm(37)* (Rv1988) (Supplementary Fig. S1a), was not observed in any other strains of the Da Nang cohort (Fig. 1a). The same deletion was reported previously in EAI strains of our northern Vietnam cohort^15^ (Fig. 1b). This deletion was also identified in EAI4_VNM and ZERO strains of the southern Vietnam cohort (Supplementary Fig. S1b and S2) and of the Asia-Africa data set^16^ (Supplementary Fig. S3), and in EAI4_VNM (SIT139, L1.1.1.1) strains of the Thai set, but it was not seen in the Philippine set where EAI4 and ZERO strains were not observed (Figures not shown).

### L1.1.1.1-specific structural variants and RD900

Using long- and short-read sequencing, followed by a hybrid assembly approach, complete genome sequences of the five L1.1.1.1 samples, two from EAI4_VNM (DN-049 and DN-105), and three from the ZERO group (DN-059, DN-068 and DN-101), were obtained. Comparison with H37Rv using NucDiff^24^ revealed that they all had the 2,153 bp sequence known as the Mtb-specific deletion 1 region (TbD1), which is deleted in L2 to L4, modern lineages^25^. Two L1.1.1.1-sublineage-specific deletions longer than 50 bp, one in *PE_PGRS4* (1,146 bp), and the other in *PE_PGRS22* (189 bp), were further identified when comparing the publicly available L1.1.1 complete genome sequence (CP041795.1) (Supplementary Fig. S4a and b). Out of the 838 deduced amino acids of *PE_PGRS4* in H37Rv, 382 amino acids, including the second GRPLI motif, were lost by the deletion (Supplementary Fig. S4c). The 78 L1.1.1.1 strains exclusively carried this deletion when evaluated by the alignment of short reads of all samples in this study (Figure not shown).

According to the original report^26^, RD900 was absent in the modern type of Mtb strains but was present in the L6 Mtb West African 2, *Mycobacterium africanum*. This region was also present in the complete genome of our L1.1.1.1 (EAI4_VNM or ZERO) strains (Supplementary Table S1, Supplementary Fig. S5a). The RD900 locus had two ORFs that coded for a putative ABC transporter ATP-binding protein and PknH2 (Supplementary Fig. S5b and c)^26^. *PknH2* of L1 strains had the same length as those of the *Mycobacterium tuberculosis* variants *bovis* and *canettii*, whereas their sequences were longer than that of L6 Mtb West African 2 by 21 amino acids (Supplementary Fig. S5c). When the complete genome sequences of L1 strains deposited by others were analyzed together, the whole 4,381 bp region spanning the RD900-specific sequence (3,141 bp) was deleted in 7 of the 28 L1 genomes, which was not limited to particular L1 sublineages (Supplementary Table S2). Two L1.1.3 strains had a 90-bp deletion in the proline-rich region of *PknH1* accompanied by the intact RD900 region.

The BLAST-based RepUnitTyping tool (https://github.com/NKrit/RepUnitTyping) was utilized for searching the presence or absence of the RD900 locus from the short-read data with a multifasta file made up of six sequences specific to the ABC transporter and *pknH2* genes, together with six sequences in adjacent genes as controls (Supplementary Table S3a). The short reads specific to the region were lacking in all L2 and L4 strains, which indicated the RD900 deletion, whereas they were also missing in some of the L1 strains (Fig. 1a,b). The deletion was observed independently of L1 sublineages in Da Nang and in other geographical areas, although no deletion was observed in a majority of the strains; L1 strains showing the deletion accounted for 8.0% (7/87) in our study, and 17.1% (14/82), 7.9% (31/391), 14.6% (70/480), and 4.9% (7/144) in the northern Vietnam, southern Vietnam, Thai, and Philippine studies, respectively.

### Genetic variants specific to ZERO strains

After correction of multiple comparisons using Fisher’s exact test, we found five genetic loci harboring deletions that were significantly associated with ZERO strains (Supplementary Table S4a). Of these associations, a 118 bp deletion in *furA* (Rv1909c) (Supplementary Fig. S6a) was most significant (*P* = 2.130E−15, Supplementary Table S4a). This deletion was found in all strains of the ZERO clade and was not seen in any other clades, including EAI4_VNM, in our study (Fig. 1a).

We further confirmed the uniqueness of the *furA* deletion in five comparison data sets. In the southern Vietnam and the Asia-Africa data sets^16^, all ZERO strains had this deletion (Supplementary Fig. S2, S3, and S6b). In the Thai^18^ and the Philippine sets^27^, where the ZERO clade was not present, this deletion was not observed (Figures not shown).

According to a Fisher’s exact test, 49 SNVs were found to be significantly associated with ZERO strains, and the most significant SNVs indicated V1A in Rv0826 and A136A in *fadE27* (Rv3505) (corrected *P* value = 2.13E−14; Supplementary Table S4b). These two SNVs were exclusively seen in the ZERO-clade strains (Fig. 1a).

We also confirmed the specificity of these two SNVs in Rv0826 and *fadE27* in the comparison data sets. They were found only in the ZERO strains of southern Vietnam (Supplementary Fig. S2) and the Asia-Africa data sets (Supplementary Fig. S3), but were not found in the nonZERO strains in other countries (Figures not shown).

### No copies of IS*6110* in the ZERO strains

Screening of short-read-sequencing data by RepUnitTyping suggested that all ZERO spoligotype strains did not have traces of any IS*6110* copies, whereas the nonZERO strains had at least one copy. Three full genome sequences of ZERO strains did not have any IS*6110* elements (Supplementary Fig. S7). Structural variants identified with long-read analysis are summarized in Supplementary Table S1. Consequently, not only the 118 bp deletion in *furA*, but also a 3,359 bp region of the CRISPR sequences was specifically deleted in the ZERO strains. Because this direct repeat region harbors one intervening IS*6110* element, which was assumed to be the original insertion site in the genome of *Mycobacterium tuberculosis* complex^28^, it was also lost in the ZERO strains and, subsequently, no IS*6110* elements were left there.

Among the five comparison data sets, a lack of IS*6110* sequences was seen in all ZERO spoligotype strains and four nonZERO strains of southern Vietnam, in ZERO strains from Asia-Africa, and in one unidentified spoligotype strain from Thailand. This absence was not seen in any of the northern Vietnam or the Philippine sets (Figures not shown).

## Discussion

Our study demonstrated that EAI Mtb strains, especially those of a typical Vietnamese EAI4_VNM and a pattern similar to EAI4_VNM (EAI4_VNM-like), were most frequently observed in Da Nang City and that ZERO strains showing a distinctive spoligo-pattern were genetically closely related to the EAI4 clade in Vietnam. All of them were found in the L1.1.1.1 sublineage defined by the updated SNP-based barcode^5^. A deletion in RD2bcg spanning six loci, i.e., *PE_PGRS35*, *cfp21*, Rv1985c, Rv1986, Rv1987, and *erm(37)*, was found to be specific to the above EAI4_VNM and ZERO strains, whereas another 118 bp deletion in *furA* and two SNVs were specific to ZERO strains only. These genomic characteristics were not found in the non-Vietnamese L1 strains in Thailand, the Philippines and other areas, but were unique to our L1 subgroup. RD900, another ancestral marker, was detected in most of the L1 strains, but was not always found in our cohort and other data sets. Mtb strains without IS*6110* copies accounted for 5.0% and all had the ZERO spoligotype in our cohort. Clinical Mtb isolates belonging to the Vietnamese L1 subgroup were phylogenetically close to each other but were not too close to suspect direct transmission as defined by ≤ 12 SNV differences. Patients infected with ZERO strains had less lung infiltration compared with other strains.

The predominance of EAI over the Beijing sublineage in middle Vietnam, and vice versa in northern and southern Vietnam, were consistent with a previous report^14^. We demonstrated that the Vietnamese L1.1.1.1 consisting of EAI4_VNM, ZERO, and some EAI5 strains were associated with older age at onset and that clustered strains indicating recent spread were rare. This suggests possible replacement of indigenous EAI strains by more modern lineages. Currently, some L4 strains might be actively transmitted in Da Nang. Signs of recent spread of L2/Beijing strains were not clear in Da Nang, whereas they were remarkable in Hanoi, a more urban area in Vietnam, which was consistent with a previous report^14^. Drug resistance was highly observed only in L2/Beijing strains in both cohorts. Spread patterns of Mtb strains in Vietnam are of global concern because TB among Vietnam-born migrants is often a public health problem in low TB-burden countries^29–31^, and also because outbound education and career development have recently been more common among the Vietnamese.

The deletion size in RD2bcg that was observed in EAI4_VNM and ZERO strains was smaller than that in the original BCG strain (5,992 bp vs. 10,701 bp) and was not observed in the EAI5 group, suggesting that this is a specific genomic marker that differentiates a subgroup inside Vietnamese L1.1.1.1^15^. In ZERO strains, in addition to the RD2bcg deletion, a unique 118 bp deletion disrupting *furA* was found, suggesting that ZERO strains evolved from the EAI4_VNM clade. Both types of strains appear unique to Vietnam or Vietnamese descents, although the evolutionary process of these genotypes needs to be clarified further in future large-scale investigations of Mtb genotypes in the neighboring southeast Asian countries Cambodia and Laos.

The original RD2 locus, which encodes 11 ORFs from Rv1978 to Rv1988, was reported to contribute to Mtb virulence^32^. Some of antigens, Rv1983 (PE_PGRS35), Rv1984c (cfp21), and Rv1985c to Rv1987, encoded by RD2 genes, harbor T- and/or B-cell epitopes^33^ and are highly variable, suggesting a role for genetic variation in evading host immunity^34^. CFP21, a RD2 secretory protein, could play an important role in Mtb pathogenesis by disrupting the host alveolar barrier and thereby facilitating mycobacterial dissemination^35^. RD2 is likely to have been an unstable region during evolution, as it can be lost from Mtb during passage. Deletion of multiple genes in this area may result in less virulent Mtb^32^ and RD2-absent BCG, which indeed leads to a less immunogenic vaccine, though the protection against pulmonary disease was not affected in a murine model^36^. In our study, the EAI4_VNM and ZERO strains harboring a deletion in the RD2 locus were also lacking Rv1983 to Rv1987.

In the Mtb genome, *furA* is located upstream of *katG*, and the two genes constitute an operon that could be co-transcribed from a common regulatory region upstream of *furA*^37^. Truncation of the operon’s upstream area can confer high level resistance to INH^38^. FurA could regulate genes, other than *katG*, that are involved in pathogenesis^37^.

When analyzing the complete genome assembled using long- and short-read sequences, L1.1.1.1-specific deletions (> 100 bp) were identified in PE_PGRS genes. *PE_PGRS4* is one of the four PE_PGRS genes with two GRPLI motifs^39^, and the large deletion spanning the second GRPLI motif is likely to cause structural alteration of the protein. In addition, large insertions, such as TbD1 and RD900, were detected. Because the RD900 locus contains repetitive sequences, long reads were necessary to determine the exact location. RD900 was originally reported as a marker for the *Mycobacterium tuberculosis* variant *africanum*, and is lacking in modern Mtb lineages L2 to L4^26,40^. In our cohort, as shown in the analyzed panels, the intact RD900 was observed in most of the L1 strains, but it was deleted in some (5% to 15%) of the strains, presumably because the region is unstable and is often lost due to homologous recombination in the flanking regions and is non-specific to the lineage. In addition, complete genomes of two L1.1.3 strains in a public database had another deletion in the proline-rich region of *PknH1*. This deletion has been observed only in animal-adapted and L5/L6 strains and was reported to be associated with virulence of *M. bovis*^40^. When more complete genome sequences of L1 strains are deposited in the future, the sublineage dependency of these deletions and their host–pathogen relationship should be further tested. Genes located in the RD900 locus, including a putative ABC transporter, are interesting and might have been necessary in the ancestral environment before Mtb became a human pathogen, but their functions have not been studied, because these genes are never recognized as long as H37Rv, an L4 strain, is used as a reference genome of short-read mapping.

IS*6110* is a transposable element of which the copy number in the genome and transposable activity can generate genotypic variation^41^. Mtb strains with no copies of IS*6110* were reported in 1993^42^. Although they were originally found in Asian immigrants with TB mainly living in the USA or France^43^, Mtb strains without IS*6110* have also been found in other regions; ranging from 5.1% to 23.8% depending on the areas of India^44–47^, 4.1% in the rural area of southern Vietnam^48^, 1.9% in the city areas of the south^21^ (estimated by our analysis), and 5.0% in our study. The strains harboring no IS*6110* DNA usually belong to ancestral lineages, including L1^28^, although in our cohort, no copies of IS*6110* were only found in ZERO strains. This deserves consideration because TB diagnostic tests detecting the IS*6110* sequence are useless for these strains^49^.

The presence of a moderate copy number of IS*6110* can provide a selective advantage toward bacterial virulence^41^. It is well known that the Mtb Beijing lineage harbors a high copy number of IS*6110*^50^ and is associated with high virulence, extensive drug resistance, and transmission^51^. Mtb strains without IS*6110* have been reported to be less drug resistant^48^ and are also associated with less infiltration on chest X-rays than others in our study. Nevertheless, it is not known whether there is a causal relationship between the IS*6110* copy number and pathogenic behaviors; drug resistance and outbreak has also happened with low copy number strains^41^. Although the presence of these elements and their transposition, including insertion sites, are important for the evolution of the Mtb genome^28^, their true influence on bacterial fitness and successful adaptive evolution remains controversial.

Our study has some limitations. First, our sample number was less than two hundred in the central area of Vietnam, which is smaller than reported in other larger studies. Nevertheless, our meta-data have less bias, because they were obtained in a prospective manner from a population-based study in a central area of Vietnam. Our study findings were also confirmed by comparing other data sets in the public database, including the northern and southern areas of the country and in other Asian and African countries. Second, we were not able to conduct *in-vitro* experiments to demonstrate the functional significance of newly identified structural variants that disrupt genes with unknown roles in these Vietnamese strains, although this was not in the scope of our study due to resource limitations.

In conclusion, we characterized an L1 subgroup, i.e., L1.1.1.1, which included the distinctive and a well-reported Vietnamese EAI4_VNM and a rare ZERO spoligotype that was presumably evolved from EAI4_VNM. Specific structural variants, large deletions spanning many genes, were identified in the genome. Analyses of age at onset and genotypic clusters through our cohorts suggested that transmission of these L1.1.1.1 strains might be rather inactive, compared with other modern lineages. All ZERO strains that were analyzed did not have any IS*6110* elements, and patients infected with these strains had an association with less lung infiltration. Characterization of the ancestral L1 lineage is useful for TB management in Asia and Africa and also provides a basis to understand virulence and evolutionary processes shifting from ancestral to modern Mtb lineages, and to consider countermeasures against the pathogen throughout the world.

## Methods and materials

### Study sites, patient recruitment, and sample collection

Patients who were over 18 years of age and were diagnosed with smear-positive pulmonary TB were recruited from all district TB centers and Da Nang Lung Hospital. After providing informed consent, patients were interviewed using a structural questionnaire, and sputum samples were collected for culture and drug susceptibility testing. Clinical information was collected from medical records and chest X-rays.

### Ethics statement

The study was approved by the Ethics Committee for Biomedical Research, National Hospital of Pediatrics, Vietnam, and the Research Institute of Tuberculosis, Japan Anti-Tuberculosis Association, Japan. All experiments were performed in accordance with relevant guidelines and regulations.

### WGS analyses using short reads

The DNA extraction method was described previously^16^. For short-read WGS, libraries were prepared with a QIAseq FX DNA Library Kit (QIAGEN) and paired-end sequencing (350 bp for read1 and 250 bp for read2) was performed using MiSeq (Illumina). Raw sequence data for the Mtb strains were deposited in the DRA database under the accession number DRA011280. Mapping was performed using bwa-mem v0.7.15 to the complete genome sequences of H37Rv (AL123456.3) and an EAI4_VNM strain in Hanoi reported by our group (AP018033.1)^52^ when necessary. Phylogenetic trees were constructed with the maximum likelihood method using RAxML version 8.2.8 (https://github.com/stamatak/standard-RAxML) and then visualized with plotTree (https://github.com/katholt/plotTree) using *Mycobacterium canettii* (ERR313114) as an out-group. Genetic clusters were defined by the pairwise differences of no more than 12 SNVs using in-house python scripts.

Short-read sequencing data were further subjected to *in-silico* spoligotyping using the SpoTypingv2.1-commandLine tool. Spoligotype patterns were identified based on the SITVIT2 database^53^. In the present study, spoligotype patterns characterized by the absence of spacers 26, 27, 29 to 32, and 34 and the presence of 33 in the CRISPR locus, but not registered in the database, were regarded as “EAI4_VNM-like” strains, whereas “EAI5-like” was assigned for strains in which spoligotypes showed the absence of spacers 29 to 32 and 34, but were not registered in the database. Drug resistance-conferring mutations, small indels, and lineage-specific variations were extracted using TBProfiler version 3.0.3^5^ (https://github.com/jodyphelan/TBProfiler). The Beijing genotype was further classified into ancient and modern Beijing sublineages by detecting the SNVs at the nucleotide position 649,345, which is equivalent to the presence of IS*6110* in the NTF region^54^.

RD-Analyzer 1.0^23^ was used to assess the presence or absence of 31 RDs, as well as to screen for deletions by detecting no coverage areas in the whole genome. For this deletion screening, complete genome sequences of the clinical isolates belonging to L1 or L2 in our Hanoi cohort, AP018033 to AP018036^52,55^ as well as the H37Rv genome (L4), were used as references.

We also retrieved five WGS data sets from a public database, which were analyzed for comparisons, to characterize Mtb strains in Da Nang city. The northern Vietnam data set (accession numbers: DRA008666-7 and DRA008677) provided the data, including clinico-epidemiological information of 332 samples in Hanoi city, in the north of Vietnam, which was reported previously by our group^15,20^. The southern Vietnam set (accession number PRJNA355614) included 1,635 isolates collected in Ho Chi Minh city in the south of Vietnam^21^. The Thai set (accession numbers: ERR718196-ERR846998) included 480 L1 strains collected in a northern province of Thailand^18^. The Philippine set (accession number ERP110368) included 178 strains collected in the Philippines^27^. The Asia-Africa set included 43 strains collected from other Asian and African countries^16^. Also, 22 complete genome sequences of L1 strains available in the public database were downloaded (Supplementary Table S2).

### Detection of IS*6110* elements

A multifasta file was made with seven sequences specific to IS*6110* and six sequences from essential genes as positive controls (Supplementary Table S3b). The non-prediction mode of RepUnitTyping version 1.5 (https://github.com/NKrit/RepUnitTyping) incorporating a BLAST search function across the entire reads was used to assess the presence or absence of IS*6110* elements, since the prediction mode was not appropriate to count the IS*6110* copy number from the short reads using the library kit including the PCR amplification process^15^.

### Genome assembly using long- and short-sequencing reads

The three best samples of ZERO strains in terms of DNA quality and quantity were subjected to long-read sequencing, together with two EAI4_VNM and four L2/Beijing strains. Libraries were prepared from 1 μg of DNA using a SQK-LSK109 kit (Oxford Nanopore Technologies, Oxford, UK) following the manufacturer’s protocol, except for the incubation time for nick-repair, and end-prep was increased to 20 min. GridION sequencing was performed using FLO- MIN106D. Raw sequence data for these Mtb strains were deposited in the DRA database under the accession number DRA011281.

Flye version 2.8.3 with POLCA in MaSuRCA v3.4.1 was applied for genome assembly^56,57^ (https://github.com/fenderglass/Flye; https://github.com/alekseyzimin/masurca) using long- and short**-**read data, and when ambiguous sequences were obtained, another hybrid assembly tool, Unicycler^58,59^ version 0.4.8 (https://github.com/rrwick/Unicycler), was used for confirmation. The complete genome sequences have been deposited in the DDBJ/ENA/GenBank under the accession numbers AP024454–AP024462. NucDiff version 2.0.3 was used for whole genome alignment to compare assembled sequences with a reference genome^24^. A query sequence, X17348, was further prepared to determine the copy number of IS*6110* in the assembled sequences using Bandage version 0.8.1 (https://github.com/rrwick/Bandage).

### Association analyses

Chi-squared and Fisher’s exact tests were performed to compare the frequencies of events among the groups. The Cochran–Armitage test for trend was used to analyze age-dependent frequencies of events. Bonferroni’s correction was applied for multiple comparisons. Wilcoxon’s rank-sum test was used to compare non-parametric distributions between the groups. Possible associations between given Mtb sublineages and genetic variants or phenotypic characteristics, adjusted for patients’ age, gender, and BMI, were further studied using logistic regression models. These analyses were performed using STATA version 16 (StataCorp LLC, College Station, TX, USA), and P values less than 0.05 were considered statistically significant.

## Supplementary Information

Supplementary Information.

## Data Availability

All data pertaining to the manuscript have been provided in the forms of tables and figures. Supporting information is available as Supplementary Tables S1–S4 and Supplementary Figures S1–S7. Datasets pertaining to the sequence searches described here are available from the corresponding author on request.
