# Supplementary material for: Phenotypic and genotypic features of the Mycobacterium tuberculosis lineage 1 subgroup in central Vietnam
Source: Sci Rep. 2021 Jun 30;11:13609. doi: 10.1038/s41598-021-92984-5 (PMC8245516; doi:10.1038/s41598-021-92984-5)

Supplementary Information

Phenotypic and genotypic features of the *Mycobacterium tuberculosis* lineage 1 subgroup in central Vietnam

Nguyen Thi Le Hang[1,11], Minako Hijikata[2,11], Shinji Maeda[3], Akiko Miyabayashi[2], Keiko Wakabayashi[2], Shintaro Seto[2], Nguyen Thi Kieu Diem[4], Nguyen Thi Thanh Yen[4], Le Van Duc[5], Pham Huu Thuong[6], Hoang Van Huan[6], Nguyen Phuong Hoang[7], Satoshi Mitarai[8], Naoto Keicho[9,10*] Seiya Kato[9]

[1] NCGM-BMH Medical Collaboration Center, Hanoi, Vietnam.
[2] Department of Pathophysiology and Host Defense, The Research Institute of Tuberculosis, JATA, Tokyo, Japan.
[3] Faculty of Pharmaceutical Sciences, Hokkaido University of Science, Hokkaido, Japan.
[4] Department of Microbiology, Da Nang Lung Hospital, Da Nang, Vietnam.
[5] Da Nang General Hospital, Da Nang, Vietnam
[6] Hanoi Lung Hospital, Hanoi, Vietnam.
[7] Department of Microbiology, Hanoi Lung Hospital, Hanoi, Vietnam.
[8] Department of Mycobacterium Reference and Research, The Research Institute of Tuberculosis, JATA, Tokyo, Japan.
[9] The Research Institute of Tuberculosis, JATA, Tokyo, Japan.
[10] National Center for Global Health and Medicine, Tokyo, Japan.
[11]These authors contributed equally to this work.

***Corresponding author**
Naoto Keicho, MD, PhD
Vice Director
The Research Institute of Tuberculosis
Japan Anti-Tuberculosis Association
3-1-24 Matsuyama, Kiyose, Tokyo 204-8533, JAPAN
E-mail: nkeicho@jata.or.jp

Table and figure legends

Supplementary Table S1. Insertions/deletions identified through long-read analysis, larger than 50 bp between EAI4_VNM HN-024 strain (AP018033.1) and the ZERO strains.

Insertions/Deletions identified through long-read analysis that were larger than 50 bp when compared to AP018033.1, and shared by all of the three ZERO strains, are listed. In the deletion type, position numbers indicate breakpoints in each strain. Positions of the insertion/deletion variant that distinguishes ZERO strains from others are not specified (=NA) in Beijing strains, because they have a larger deletion (spacer 1–34 in the direct repeat locus of the CRISPR sequences), spanning the above variants. Differences in tandem repeats, that is copy numbers of VNTR (MIRU2, locus 0595, MIRU 10, QUB 11b and QUB 15), are not included in the list.

Ins: insertion or presence; Del: deletion or absence; NA: not applicable.

Variant in bold: specific to ZERO strains.

Supplementary Table S2: Presence or absence of the RD900 region in L1 complete genome sequences available in our previous and current studies (n = 6) and reported by others in a public database (n = 22).

The presence (P) or absence (A) of the 4,381-bp sequence at the RD900 region[26] and of the 90-bp proline-rich region in pknH1/2[40] were detected by the BLAST-based search.

*Sublineages of L1 strains were determined using TBProfiler v3.0.3[5].

**Wada T, *et al.* [52].

NA: not assessed because *pknH2* itself is absent due to the RD900 deletion.

Supplementary Table S3: Multifasta files used for a BLAST search incorporated in RepUnitTyping (https://github.com/NKrit/RepUnitTyping).

a) for identification of RD900

b) for identification of IS*6110**

*IS*6110* sequences were extracted from the complete genome of eight Mtb strains belonging to L1 (AP018033.1), L2 (AP018034.1, AP018035.1 and AP018036.1) and L4 (AL123456.3, NC_002755.2, NC_020559.1, AP014573.1), and seven sets of 50-nt sequences that were exactly identical to each other were selected as references to identify the presence or absence of IS*6110* using RepUnitTyping. Additional six nucleotide sequences from essential genes were selected as positive controls.

Supplementary Table S4: Genetic variants specific to ZERO strains

a) Deletions significantly associated with the ZERO strains*.

b) Single nucleotide variants (SNVs) significantly associated with the ZERO strains**.

*Bonferroni's correction was applied for multiple comparisons, and $P < 1.084E-05$ was regarded as significant.

**Bonferroni's correction was applied for multiple comparisons, and $P < 2.581E-06$ was regarded as significant.

Del: deletion.

Supplementary Fig. S1: A large deletion spanning *PE_PGRS35*, *cfp21*, Rv1985c, Rv1986, Rv1987, and *erm(37)* was observed in ZERO, EAI4_VNM and EAI5 strains in Da Nang (a) and southern Vietnam (b), viewed by Integrative Genomics Viewer (IGV) version 2.3.88 (http://software.broadinstitute.org/software/igv/home).

Supplementary Fig. S2: Phylogenetic tree of 1,635 strains of the southern Vietnam data set, constructed with the maximum likelihood method using RAxML version 8.2.8 (https://github.com/stamatak/standard-RAxML) and visualized with plotTree for python v2.7 (https://github.com/katholt/plotTree). RD239, RD2bcg, deletion in *furA*, and SNVs in correlation with Mtb clades are shown. Mtb: *Mycobacterium tuberculosis*, SNV: single nucleotide variant, Del: deletion.

Supplementary Fig. S3: Phylogenetic tree of 43 lineage 1 strains from the Asia-Africa data set, constructed with the maximum likelihood method using RAxML version 8.2.8 (https://github.com/stamatak/standard-RAxML) and visualized with plotTree for python v2.7 (https://github.com/katholt/plotTree). RD239, RD2bcg, deletion in *furA*, and SNVs in correlation with Mtb clades are shown. Mtb: *Mycobacterium tuberculosis*, SNV: single nucleotide variant, Del: deletion.

Supplementary Fig. S4: Structural variants of L1.1.1.1.
a) A structural variant of L1.1.1.1 in *PE_PGRS4*.
PE_PGRS4 (Rv0279c) nucleotide sequences of H37Rv (AL123456.3), CP041795 (L1.1.1), and DN-049 (AP024454, L1.1.1.1) were aligned by Genetyx-Mac ver.21 (GENETYX Corporation, Tokyo, Japan). Dots and dashes represent identical and deleted nucleotides, respectively.
b) A structural variant of L1.1.1.1 in *PE_PGRS22*.
PE_PGRS22 (Rv1091) nucleotide sequences of H37Rv (AL123456.3), CP041795 (L1.1.1), and DN-049 (AP024454, L1.1.1.1) were aligned by the ClustalW module built into Genetyx-Mac ver.21 (GENETYX Corporation, Tokyo, Japan). Dots and dashes represent identical and deleted nucleotides, respectively.
c) Alignment of deduced amino acid sequences of PE_PGRS4 (Rv0279c) for H37Rv (AL123456.3), CP041795 (L1.1.1), and DN-049 (AP024454, L1.1.1.1) using Genetyx-Mac ver.21 (GENETYX Corporation, Tokyo, Japan). PE_PGRS4 is known to have two GRPLI motifs[39], and the second one within the PGRS domain is lost due to the 382-amino-acid deletion in L1.1.1.1. Dots and dashes represent identical and deleted amino acids, respectively.

Supplementary Fig. S5: Comparison of the RD900 region.
a) Nucleotide sequence alignment of the RD900 region. Nucleotide sequences from *pknH1* to *pknH2* of L1 Mtb DN-059 (Accession no. AP024455 in this study), Maf_GM041182 (L6 Mtb West African 2 or *Mycobacterium africanum* strain GM041182, NC_015758.1), and H37Rv (AL1234556.3) were aligned by the ClustalW module built into Genetyx-Mac ver.21 (GENETYX Corporation, Tokyo, Japan).

b) c) Alignment of deduced amino acid sequences of the putative ABC transporter ATP-binding protein (b) and PknH2 (c) for L1 Mtb DN-059 (Accession no. AP024455, this study), Maf_GM041182 (L6 Mtb West African 2, or *Mycobacterium africanum*, strain GM041182, NC_015758.1), *Mycobacterium tuberculosis* variant *bovis* (Mb3601, LR699570.1), and *Mycobacterium tuberculosis* variant *canettii* (CIPT 140010059, NC_015848.1) using the ClustalW module built into Genetyx-Mac ver.21 (GENETYX Corporation, Tokyo, Japan). bovis: *Mycobacterium tuberculosis* variant *bovis*, canettii: *Mycobacterium canettii*. Dots and dashes represent identical and deleted amino acids, respectively.

Supplementary Fig. S6: A 118-bp deletion in *furA* and ZERO-clade strains among the Da Nang (a) and southern Vietnam data sets (b), viewed by Integrative Genomics Viewer (IGV) version 2.3.88 (http://software.broadinstitute.org/software/igv/home).

Supplementary Fig. S7: Genome assembly graphs of ZERO (a), EAI4_VNM (b), and Beijing (c) strains visualized with Bandage version 0.8.1 (https://github.com/rrwick/Bandage) and the distribution of IS*6110* copies detected with a BLAST search with the X17348 sequence as the query. Red triangles ▶: location of IS*6110* elements.

Supplementary Table S1. Insertions/deletions identified through long-read analysis, larger than 50 bp between EAI4_VNM HN-024 strain (AP018033.1) and the ZERO strains

Lineage	spoligotype	sample ID	Structural variants	Rv0386, Rv0387c, PPE9	Rv1264	ABC transporter ATP–binding protein, hypothetical protein PknH (Mycobacterium bovis AF2122/97); RD900	*furA*	helY–tatC intergenic region	The Direct Repeat locus of the CRISPR sequences	PPE46, PE27A, *esxR*, *esxS* , PPE47	PE_PGRS49	PPE55	*rsmA*
			Length (bp)	2251	53	4381	118	68	3359	2437	267	1761	300
L1	ZERO	DN-059	Ins/Del	Del	Ins	Ins	**Del**	Ins	**Del**	Ins	Ins	Ins	Ins
		AP024455	Nucleotide position	468179 – 468180	1411571 – 1411623	1414373 – 1418753	2154366 – 2154367	2354556 – 2354623	3109393 – 3109394	3365374 – 3367810	3727602 – 3727868	3740929 – 3742689	4391653 – 4391952
L1	ZERO	DN-068	Ins/Del	Del	Ins	Ins	**Del**	Ins	**Del**	Ins	Ins	Ins	Ins
		AP024457	Nucleotide position	468171 – 468172	1411584 – 1411636	1414386 – 1418766	2154733 – 2154734	2354923 – 2354990	3109809 – 3109810	3365619 – 3368055	3727847 – 3728113	3740907 – 3742667	4385064 – 4385363
L1	ZERO	DN-101	Ins/Del	Del	Ins	Ins	**Del**	Ins	**Del**	Ins	Ins	Ins	Ins
		AP024458	Nucleotide position	468171 – 468172	1411584 – 1411636	1414386 – 1418766	2154733 – 2154734	2354923 – 2354990	3109809 – 3109810	3365619 – 3368055	3727847 – 3728113	3740907 – 3742667	4385064 – 4385363
L1	EAI4_VNM	HN-024	Ins/Del	Ins	Del	Del	Ins	Del	Ins	Del	Del	Del	Del
		AP018033.1	Nucleotide position	468095 – 470345	1413909 – 1413910	1416658 – 1416659	2152751 – 2152868	2352988 – 2352989	3107802 – 3111160	3367196 – 3367197	3726987 – 3726988	3740047 – 3740048	4389693 – 4389694
L1	EAI4_VNM	DN-049	Ins/Del	Del	Ins	Ins	Ins	Ins	Ins	Ins	Ins	Ins	Ins
		AP024454	Nucleotide position	468168 – 468169	1411848 – 1411900	1414650 – 1419030	2155034 – 2155151	2355272 – 2355339	3110222 – 3113580	3369617 – 3372053	3731845 – 3732111	3745121 – 3746881	4395669 – 4395968
L1	EAI4_VNM	DN-105	Ins/Del	Ins	Ins	Ins	Ins	Del	Ins	Ins	Ins	Ins	Ins
		AP024459	Nucleotide position	468126 – 470376	1413647 – 1413699	1416449 – 1420829	2157949 – 2158066	2357697 – 2357698	3112527 – 3115885	3371958 – 3374394	3734141 – 3734407	3747468 – 3749229	4397951 – 4398250
L2	Beijing	DN-067	Ins/Del	Ins	Ins	Del	Ins	Ins	NA	Ins	Ins	Del	Ins
		AP024456	Nucleotide position	466474 – 468724	1412300 – 1412351	1415100 – 1415101	2139603 – 2139720	2351491 – 2351558	NA	3365464 – 3369258	3733361 – 3733627	3746706 – 3746707	4400278 – 4400577
L2	Beijing	DN-146	Ins/Del	Ins	Ins	Del	Ins	Ins	NA	Ins	Ins	Del	Ins
		AP024460	Nucleotide position	460231 – 462481	1408980 – 1409031	1411780 – 1411781	2136065 – 2136182	2346665 – 2346732	NA	3364602 – 3367922	3737001 – 3737267	3750445 – 3750446	4396419 – 4396718
L2	Beijing	DN-181	Ins/Del	Ins	Ins	Del	Ins	Ins	NA	Ins	Ins	Del	Ins
		AP024461	Nucleotide position	464363 – 466613	1415369 – 1415420	1418169 – 1418170	2141637 – 2141754	2350881 – 2350948	NA	3367475 – 3372627	3739959 – 3740225	3753404 – 3753405	4407723 – 4408022
L2	Beijing	DN-251	Ins/Del	Ins	Ins	Del	Ins	Ins	NA	Ins	Ins	Del	Ins
		AP024462	Nucleotide position	464236 – 466486	1412123 – 1412174	1414923 – 1414924	2138242 – 2138359	2348834 – 2348901	NA	3365559 – 3369353	3735258 – 3735524	3748606 – 3748607	4402383 – 4402682
L4	T–H37Rv	H37Rv	Ins/Del	Ins	Ins	Del	Ins	Del	Ins	Ins	Ins	Del	Ins
		AL123456.3	Nucleotide position	466048 – 468296	1413086 – 1413138	1415887 – 1415888	2156330 – 2156447	2352071 – 2352072	3119592 – 3122193	3377271 – 3379707	3737768 – 3738034	3751021 – 3751022	4401010 – 4401309

Insertions/Deletions identified through long-read analysis that were larger than 50 bp when compared to AP018033.1, and shared by all of the three ZERO strains, are listed. In the deletion type, position numbers indicate breakpoints in each strain. Positions of the insertion/deletion variant that distinguishes ZERO strains from others are not specified (=NA) in Beijing strains, because they have a larger deletion (spacer 1–34 in the direct repeat locus of the CRISPR sequences), spanning the above variants. Differences in tandem repeats, that is copy numbers of VNTR (MIRU2, locus 0595, MIRU 10, QUB 11b and QUB 15), are not included in the list.

Ins: insertion or presence; Del: deletion or absence; NA: not applicable.

Variant in bold: specific to ZERO strains.

Supplementary Table S2. Presence or absence of the RD900 region in L1 complete genome sequences available in our previous and current studies (n = 6) and reported by others in public database (n = 22).

Accession no.	Sublineage*	4,381 bp sequence with RD900	Proline rich-region in *pknH1*	Proline rich-region in *pknH2*	
CP041794	L1.1.1	A	P	(NA)	
CP041795	L1.1.1	P	P	A	
CP041802	L1.1.1	P	P	A	
CP045962	L1.1.1	P	P	A	
AP018033	L1.1.1.1	A	P	(NA)	Previous study (HN024)**
AP024454	L1.1.1.1	P	P	A	This study (DN-049)
AP024455	L1.1.1.1	P	P	A	This study (DN-059)
AP024457	L1.1.1.1	P	P	A	This study (DN-068)
AP024458	L1.1.1.1	P	P	A	This study (DN-101)
AP024459	L1.1.1.1	P	P	A	This study (DN-105)
CP041792	L1.1.2	A	P	(NA)	
CP041793	L1.1.2	P	P	A	
CP041798	L1.1.2	A	P	(NA)	
CP041859	L1.1.2	P	P	A	
CP041790	L1.1.3.1	P	A	A	
CP041791	L1.1.3.1	P	A	A	
CP041800	L1.2.1.1	P	P	A	
CP041801	L1.2.1.1	P	P	A	
CP041828	L1.2.1.2	P	P	A	
CP009427	L1.2.1.2.1	P	P	A	
CP029065	L1.2.1.2.1	A	P	(NA)	
CP041826	L1.2.1.2.1	P	P	A	
CP041827	L1.2.1.2.1	P	P	A	
CP046308	L1.2.1.2.1	P	P	A	
CP041811	L1.2.2.1	A	P	(NA)	
CP003234	L1.2.2.2	A	P	(NA)	
CP041796	L1.2.2.2	P	P	A	
CP041868	L1.2.2.2	P	P	A	

The presence (P) or absence (A) of the 4,381-bp sequence at the RD900 region[26] and of the 90-bp proline-rich region in pknH1/2[40] were detected by the BLAST-based search.

*Sublineages of L1 strains were determined using TBProfiler v3.0.3[5].

**Wada T, et al.[52].

NA: not assessed because pknH2 itself is absent due to the RD900 deletion.

Supplementary Table S3: Multi-fasta files used for a BLAST search incorporated in RepUnitTyping (https://github.com/NKrit/RepUnitTyping)

a) for identification of RD900
>pknH_1_1
TCATTCCTTGTTGACTTTGTCAACGATCTTGGCGGCGATC
>pknH_1_2
CTCCTCCGCAACCGGCTGAGGCGGCTGTACCGGCTTGGGC
>pknH_1_3
GTGGCCGGCATGGTCGGAGGCGGGACGGGCTTAGGCGGCG
>ABC_1
TCACTTACGAGCTTTGCGTTGCGGCTCGATGCGTTTGAGC
>ABC_2
TTCGTCGAGGAACAACAGCGACGGTTTGGTCAGCAGTTCC
>ABC_3
GCGGTGCATTGGGGGTGATCATGTCGGCCGTGTCTGTCAT
>pknH_2_1
TTACCCGTACTTGGCCCACCAGTTGTGCAGATCCTCAATG
>pknH_2_2
TGGGGAGGTCGCGATGTTCCGTTTTGGGTTGTCGTCCGGT
>pknH_2_3
GTGGGCATGGTCGGCGGCTGCGCGGTTACCGCCGCGGTGC
>embR_1
CTACGTGCCGCCATGCGTCCCCGCGCTGATCTGGAACGTG
>embR_2
GCCTCGAGCTCGGCGATCACTGCGCTGGCCCGCCCACACG
>embR_3
CGAAGTCGAGCCGCTTCTCCACTGTCGCGCTACCAGCCAT

b) for identification of IS*6110**
>IS2_01
CGCCGAATTGCGAAGGGCGAACGCGATTTTAAAGACCGCGTCGGCTTTCT
>IS5_01
GGACCACGATCGCTGATCCGGCCACAGCCCGTCCCGCCGATCTCGTCCAG
>IS6_01
CGCCGCTTCGGACCACCAGCACCTAACCGGCTGTGGGTAGCAGACCTCAC
>IS8_01
GGGGATCTCAGTACACATCGATCCGGTTCAGCGAGCGGCTCGCCGAGGCA
>IS9_01
AACGGCCTATACAAGACCGAGCTGATCAAACCCGGCAAGCCCTGGCGGTC
>IS10_01
GGCCACCGCGCGCTGGGTCGACTGGTTCAACCATCGCCGCCTCTACCAGT
>IS11_01
TCCTGGGCTGGCGGGTCGCTTCCACGATGGCCACCTCCATGGTCCTCGAC
>dnaA
ACGCTCTCAGCCGCCGACTCGGACATCAGATCCAACTCGG
>dnaN
CGATTGTTGTCCGATATTACCCGGGCGTTGCCTAACAAGC
>prcA
GGCTGGCGTTCTCGGCATACGACTCTTTGAGCGCGTTGGC
>prcB_R1
GAAGATAGGTCTACAGCGGGTGTTCCAGAGAGTGAATTAA
>parB_R1
GAAGTGTCCGGGACCGGTCCGCCGATTACGACATCTGCCG
>parA
GACACACCCTCCAACGCGTAGTACTCGCATTGGATCGGGA

*IS*6110* sequences were extracted from the complete genome of eight Mtb strains belonging to L1 (AP018033.1), L2 (AP018034.1, AP018035.1 and AP018036.1) and L4 (AL123456.3, NC_002755.2, NC_020559.1, AP014573.1), and seven sets of 50-nt sequences that were exactly identical to each other were selected as references to identify the presence or absence of IS*6110* using RepUnitTyping. An additional six nucleotide sequences from essential genes were selected as positive controls.

Supplementary Table S4: Genetic variants specific to ZERO strains.

a) Deletions significantly associated with the ZERO strains*.

Gene/Locus	Number of isolates				P value
	Non-ZERO		ZERO		
	Del=No	Del=Yes	Del=No	Del=Yes	
AP018035.1HN321|01999|furA	172	0	0	9	2.13E-15
AL123456.3H37Rv|00932|citA	139	33	0	9	9.50E-07
AP018035.1HN321|03167|HN321_03166	136	36	0	9	1.89E-06
AP018033.1HN024|00410|PPE9	133	39	0	9	3.57E-06
AL123456.3H37Rv|00399|Rv0386	132	40	0	9	4.38E-06

b) Single nucleotide variants (SNVs) significantly associated with the ZERO strains**.

No	Position in AL123456.3	Number of isolates				Fisher P value	Variant's category	Variant's effect	Gene	Locus	SNV	Variant
		ZERO=No Variant=No	ZERO=No Variant=Yes	ZERO=Yes Variant=No	ZERO=Yes Variant=Yes							
1	919635	171	1	0	9	2.1302E-14	missense_variant	MODERATE	Rv0826	Rv0826	c.2T>C	p.Val1Ala
2	3924105	171	1	0	9	2.1302E-14	synonymous_variant	LOW	*fadE27*	Rv3505	c.408C>T	p.Ala136Ala
3	780304	170	2	0	9	1.17161E-13	missense_variant	MODERATE	Rv0680c	Rv0680c	c.113C>T	p.Thr38Ile
4	2137343	172	0	1	8	3.68524E-13	synonymous_variant	LOW	Rv1887	Rv1887	c.1086T>A	p.Thr362Thr
5	2954263	172	0	1	8	3.68524E-13	missense_variant	MODERATE	Rv2627c	Rv2627c	c.486T>G	p.Asn162Lys
6	1382617	172	0	1	8	3.68524E-13	missense_variant	MODERATE	*corA*	Rv1239c	c.426G>T	p.Glu142Asp
7	1896900	172	0	1	8	3.68524E-13	synonymous_variant	LOW	Rv1672c	Rv1672c	c.1308G>A	p.Pro436Pro
8	87195	172	0	1	8	3.68524E-13	upstream_gene_variant	MODIFIER	Rv0076c	Rv0076c	c.-1623G>C	nan
9	951495	172	0	1	8	3.68524E-13	stop_gained	HIGH	Rv0854	Rv0854	c.313C>T	p.Gln105*
10	1507529	172	0	1	8	3.68524E-13	missense_variant	MODERATE	*rphA*	Rv1340	c.775A>G	p.Thr259Ala
11	4284343	172	0	1	8	3.68524E-13	upstream_gene_variant	MODIFIER	Rv3814c	Rv3814c	c.-4328G>C	nan
12	2391370	167	5	0	9	4.26465E-12	missense_variant	MODERATE	*mshC*	Rv2130c	c.1090G>A	p.Val364Met
13	611741	166	6	0	9	1.06616E-11	missense_variant	MODERATE	Rv0519c	Rv0519c	c.334G>A	p.Gly112Ser
14	1061178	166	6	0	9	1.06616E-11	synonymous_variant	LOW	Rv0950c	Rv0950c	c.477G>A	p.Pro159Pro
15	4248195	172	0	3	6	1.87026E-09	missense_variant	MODERATE	*embB*	Rv3795	c.1682A>G	p.Lys561Arg
16	218654	172	0	3	6	1.87026E-09	upstream_gene_variant	MODIFIER	*sigG*	Rv0182c	c.-4514G>A	nan
17	3789935	172	0	3	6	1.87026E-09	missense_variant	MODERATE	*amiD*	Rv3375	c.1315A>C	p.Thr439Pro
18	13004	172	0	3	6	1.87026E-09	synonymous_variant	LOW	*ppiA*	Rv0009	c.537C>T	p.Ile179Ile
19	1796739	172	0	3	6	1.87026E-09	missense_variant	MODERATE	*nadB*	Rv1595	c.935C>T	p.Ser312Phe
20	4087479	171	1	3	6	1.28994E-08	upstream_gene_variant	MODIFIER	Rv3644c	Rv3644c	c.-4815_-4759delTCCCGCTGGGGTCCGCTGAGGAGCCGGGCAGTCGGACCTAGTTCGGCGACGATGCGG	nan
21	4034874	172	0	4	5	8.22914E-08	missense_variant	MODERATE	*lpqF*	Rv3593	c.523G>A	p.Asp175Asn
22	4225924	172	0	4	5	8.22914E-08	missense_variant	MODERATE	Rv3779	Rv3779	c.940G>A	p.Ala314Thr
23	1858170	141	31	0	9	5.82478E-07	missense_variant	MODERATE	Rv1648	Rv1648	c.440C>A	p.Ala147Glu
24	1716414	141	31	0	9	5.82478E-07	missense_variant	MODERATE	*mmpL12*	Rv1522c	c.1199T>G	p.Leu400Arg
25	114876	140	32	0	9	7.463E-07	missense_variant	MODERATE	*nrp*	Rv0101	c.4876C>T	p.Pro1626Ser
26	3332276	140	32	0	9	7.463E-07	missense_variant	MODERATE	*ung*	Rv2976c	c.479C>G	p.Ala160Gly
27	2238001	140	32	0	9	7.463E-07	upstream_gene_variant	MODIFIER	Rv1989c	Rv1989c	c.-4702G>A	nan
28	898783	140	32	0	9	7.463E-07	upstream_gene_variant	MODIFIER	Rv0802c	Rv0802c	c.-3155G>A	nan
29	2629359	140	32	0	9	7.463E-07	missense_variant	MODERATE	*plcB*	Rv2350c	c.961G>A	p.Val321Ile
30	3459929	140	32	0	9	7.463E-07	missense_variant	MODERATE	Rv3091	Rv3091	c.814A>G	p.Thr272Ala
31	3446677	140	32	0	9	7.463E-07	missense_variant	MODERATE	Rv3081	Rv3081	c.638C>T	p.Ala213Val
32	1353013	140	32	0	9	7.463E-07	synonymous_variant	LOW	*gpgS*	Rv1208	c.870G>A	p.Leu290Leu

No	Position in AL123456.3	Number of isolates				Fisher P value	Variant's category	Variant's effect	Gene	Locus	SNV	Variant
		ZERO=No Variant=No	ZERO=No Variant=Yes	ZERO=Yes Variant=No	ZERO=Yes Variant=Yes							
33	942756	140	32	0	9	7.463E-07	missense_variant	MODERATE	Rv0846c	Rv0846c	c.1439G>C	p.Gly480Ala
34	2328954	140	32	0	9	7.463E-07	synonymous_variant	LOW	*cobM*	Rv2071c	c.24G>T	p.Ala8Ala
35	176222	140	32	0	9	7.463E-07	missense_variant	MODERATE	Rv0149	Rv0149	c.523G>A	p.Gly175Ser
36	989858	140	32	0	9	7.463E-07	frameshift_variant	HIGH	*citA*	Rv0889c	c.3_4insT	p.Thr2fs
37	3683697	140	32	0	9	7.463E-07	missense_variant	MODERATE	*atsB*	Rv3299c	c.2267G>A	p.Arg756Gln
38	705081	140	32	0	9	7.463E-07	missense_variant	MODERATE	Rv0610c	Rv0610c	c.829G>A	p.Ala277Thr
39	1815	140	32	0	9	7.463E-07	upstream_gene_variant	MODIFIER	*dnaN*	Rv0002	c.-237_-236insG	nan
40	793897	138	34	0	9	1.20126E-06	missense_variant	MODERATE	*lldD1*	Rv0694	c.563C>T	p.Ala188Val
41	1280806	138	34	0	9	1.20126E-06	missense_variant	MODERATE	*omt*	Rv1153c	c.41C>T	p.Thr14Ile
42	1337545	138	34	0	9	1.20126E-06	synonymous_variant	LOW	Rv1194c	Rv1194c	c.969T>C	p.Ala323Ala
43	1870983	138	34	0	9	1.20126E-06	missense_variant	MODERATE	*argR*	Rv1657	c.142G>A	p.Gly48Ser
44	3722271	138	34	0	9	1.20126E-06	synonymous_variant	LOW	Rv3335c	Rv3335c	c.330G>A	p.Leu110Leu
45	3568359	137	35	0	9	1.51016E-06	missense_variant	MODERATE	Rv3197	Rv3197	c.1336G>C	p.Val446Leu
46	789485	137	35	0	9	1.51016E-06	upstream_gene_variant	MODIFIER	Rv0689c	Rv0689c	c.-74T>C	nan
47	33267	137	35	0	9	1.51016E-06	missense_variant	MODERATE	Rv0030	Rv0030	c.44G>A	p.Ser15Asn
48	685712	137	35	0	9	1.51016E-06	missense_variant	MODERATE	*yrbE2A*	Rv0587	c.584C>A	p.Thr195Asn
49	2073614	137	35	0	9	1.51016E-06	synonymous_variant	LOW	Rv1828	Rv1828	c.534C>T	p.Tyr178Tyr

*Bonferroni’s correction was applied for multiple comparisons, and P < 1.084E-05 was regarded as significant.

**Bonferroni’s correction was applied for multiple comparisons, and P < 2.581E-06 was regarded as significant.

Del: deletion

Supplementary Fig. S1: A large deletion spanning *PE_PGRS35, cfp21*, Rv1985c, Rv1986, Rv1987, and *erm(37)* was observed in ZERO, EAI4_VNM and EAI5 strains in Da Nang (a) and southern Vietnam (b), viewed by Integrative Genomics Viewer (IGV) version 2.3.88 (http://software.broadinstitute.org/software/igv/home).

a) b)

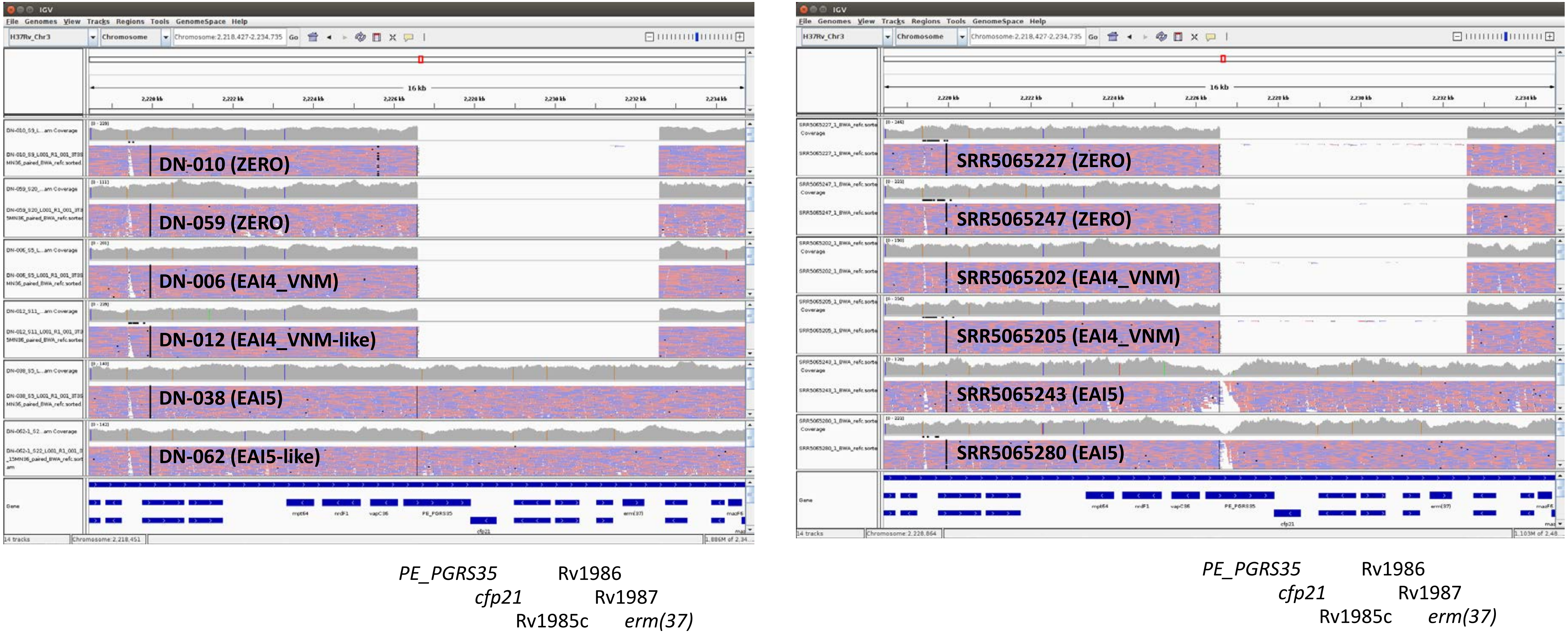

Supplementary Fig. S2: Phylogenetic tree of 1,635 strains of the southern Vietnam data set, constructed with the maximum likelihood method using RAxML version 8.2.8 (https://github.com/stamatak/standard-RAxML) and visualized with plotTree for python v2.7 (https://github.com/katholt/plotTree). RD239, RD2bcg, deletion in *furA,* and SNVs in correlation with Mtb clades are shown. Mtb: *Mycobacterium tuberculosis*, SNV: single nucleotide variant, Del: deletion

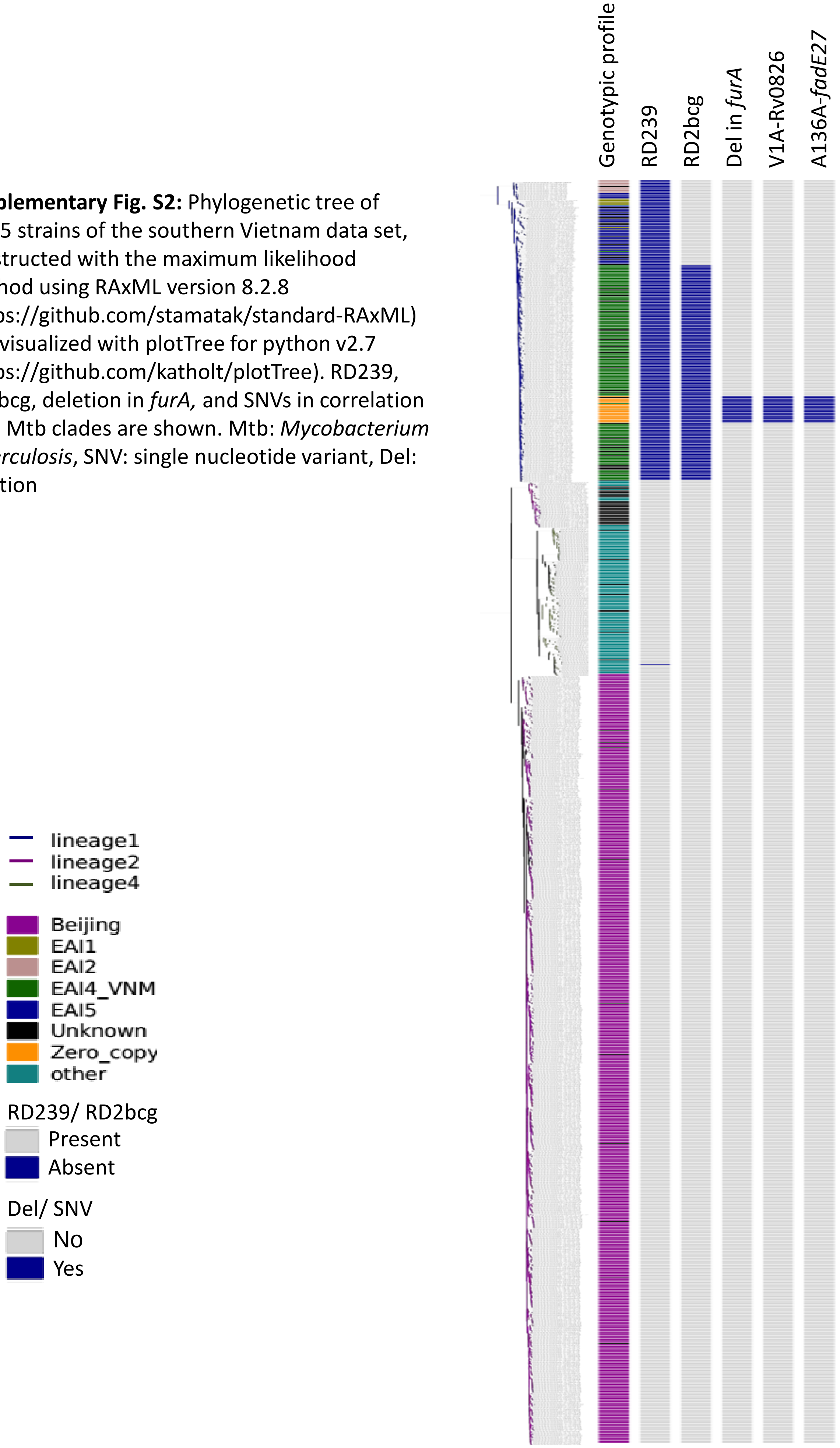

Supplementary Fig. S3: Phylogenetic tree of 43 lineage 1 strains from the Asia-Africa data set, constructed with the maximum likelihood method using RAxML version 8.2.8 (https://github.com/stamatak/standard-RAxML) and visualized with plotTree for python v2.7 (https://github.com/katholt/plotTree). RD239, RD2bcg, deletion in *furA*, and SNVs in correlation with Mtb clades are shown. Mtb: *Mycobacterium tuberculosis*, SNV: single nucleotide variant, Del: deletion.

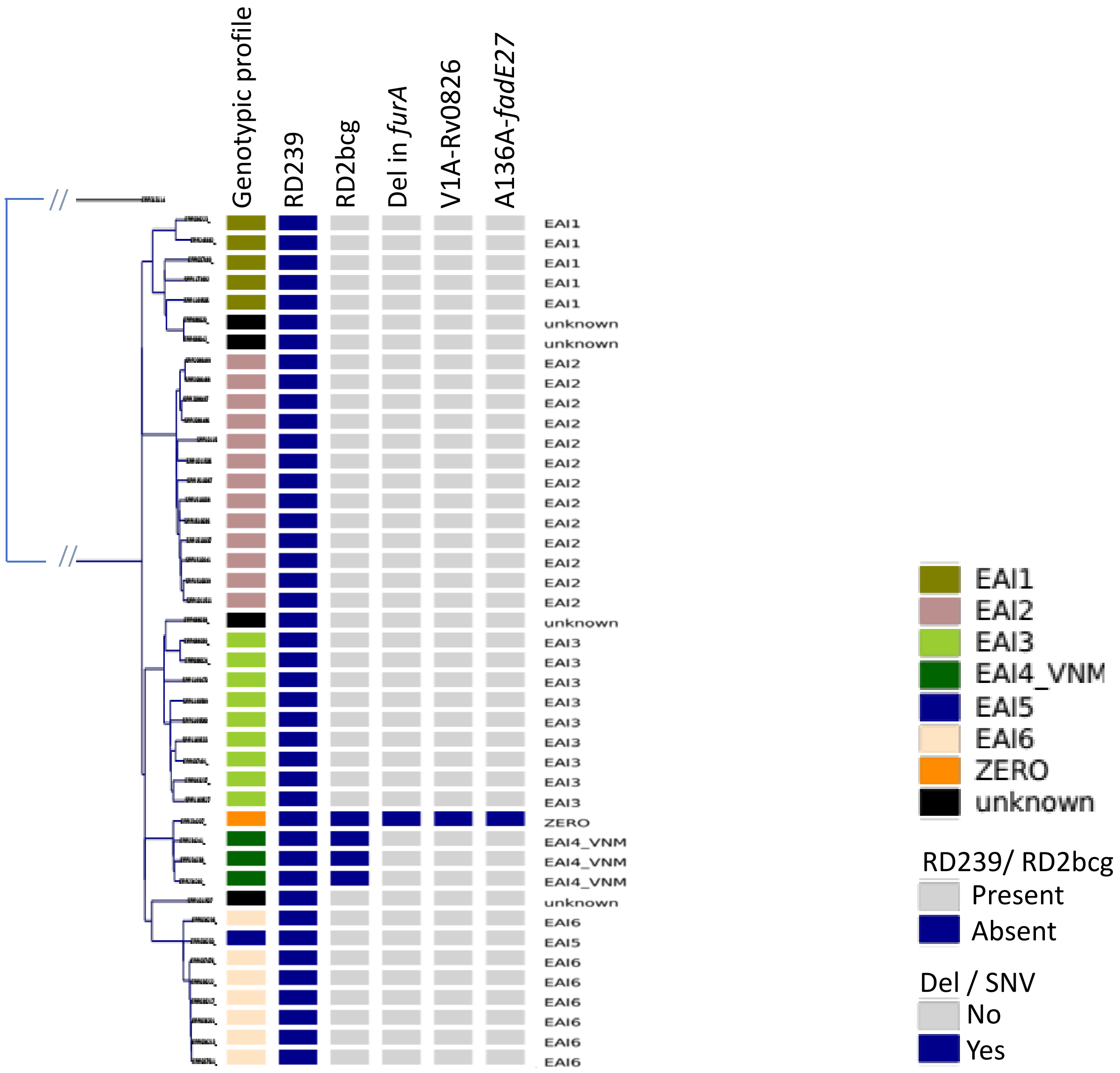

Supplementary Fig. S4 : Structural variants of L1.1.1.1

a) A structural variant of L1.1.1.1 in *PE_PGRS4*.

PE_PGRS4 (Rv0279c) nucleotide sequences of H37Rv (AL123456.3), CP041795 (L1.1.1), and DN-049 (AP024454, L1.1.1.1) were aligned by Genetyx-Mac ver.21 (GENETYX Corporation, Tokyo, Japan). Dots and dashes represent identical and deleted nucleotides, respectively.

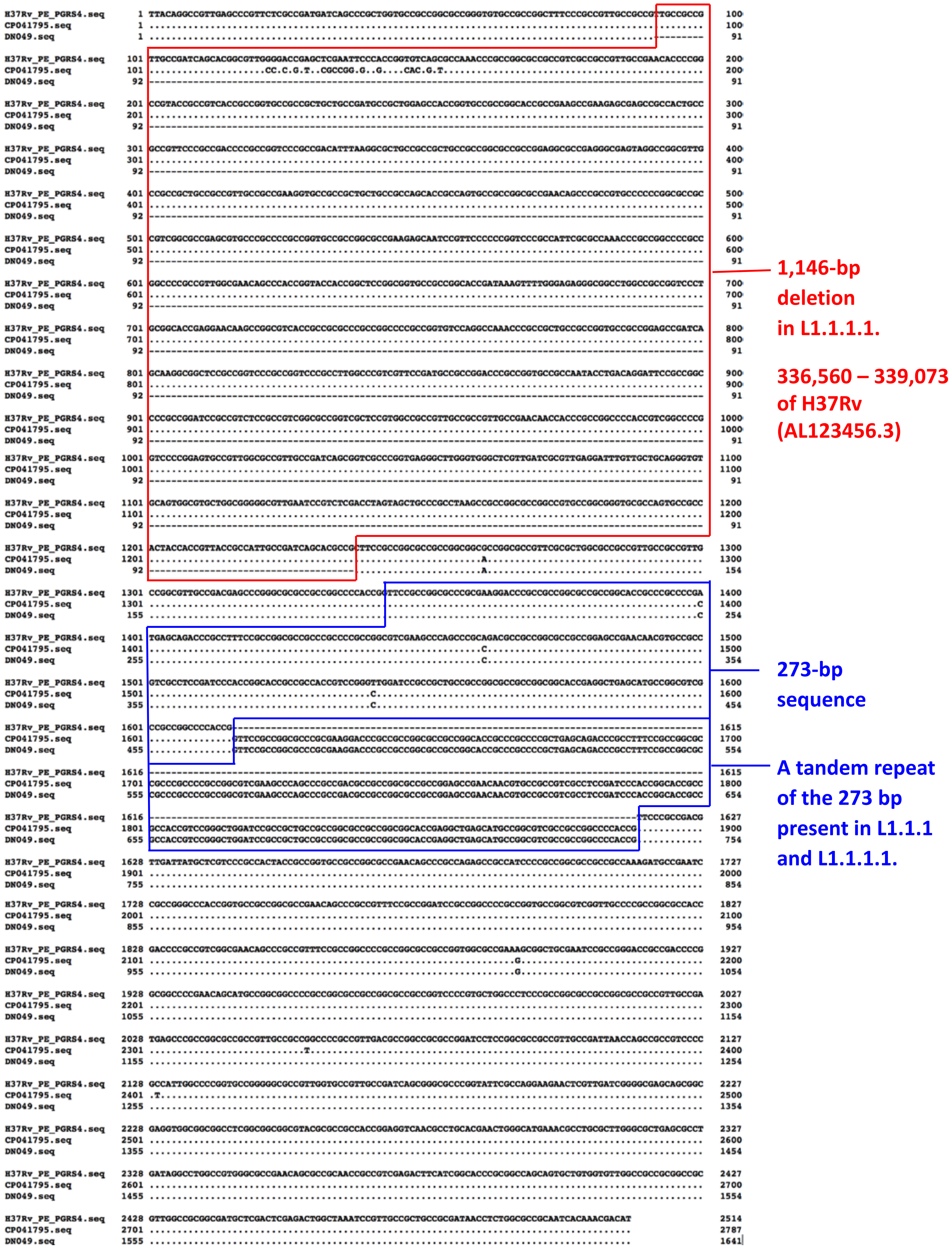

b) A structural variant of L1.1.1.1 in *PE_PGRS22*.

PE_PGRS22 (Rv1091) nucleotide sequences of H37Rv (AL123456.3), CP041795 (L1.1.1), and DN-049 (AP024454, L1.1.1.1) were aligned by the ClustalW module built into Genetyx-Mac ver.21 (GENETYX Corporation, Tokyo, Japan). Dots and dashes represent identical and deleted nucleotides, respectively.

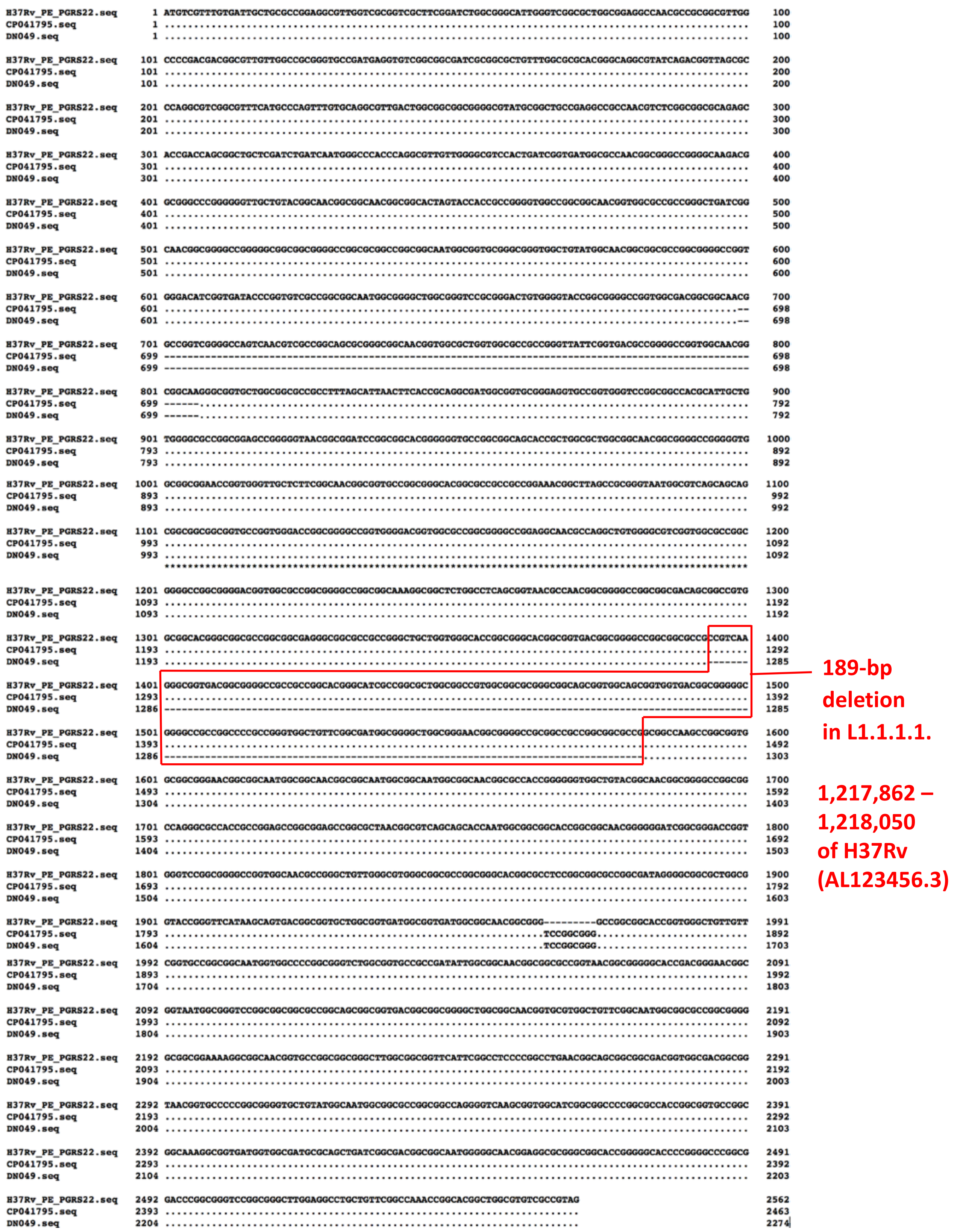

Supplementary Fig S4c): Alignment of deduced amino acid sequences of PE_PGRS4 (Rv0279c) for H37Rv (AL123456.3), CP041795 (L1.1.1), and DN-049 (AP024454, L1.1.1.1) using Genetyx-Mac ver.21 (GENETYX Corporation, Tokyo, Japan). PE_PGRS4 is known to have two GRPLI motifs[39], and the second one within the PGRS domain is lost due to the 382-amino-acid deletion in L1.1.1.1. Dots and dashes represent identical and deleted amino acids, respectively

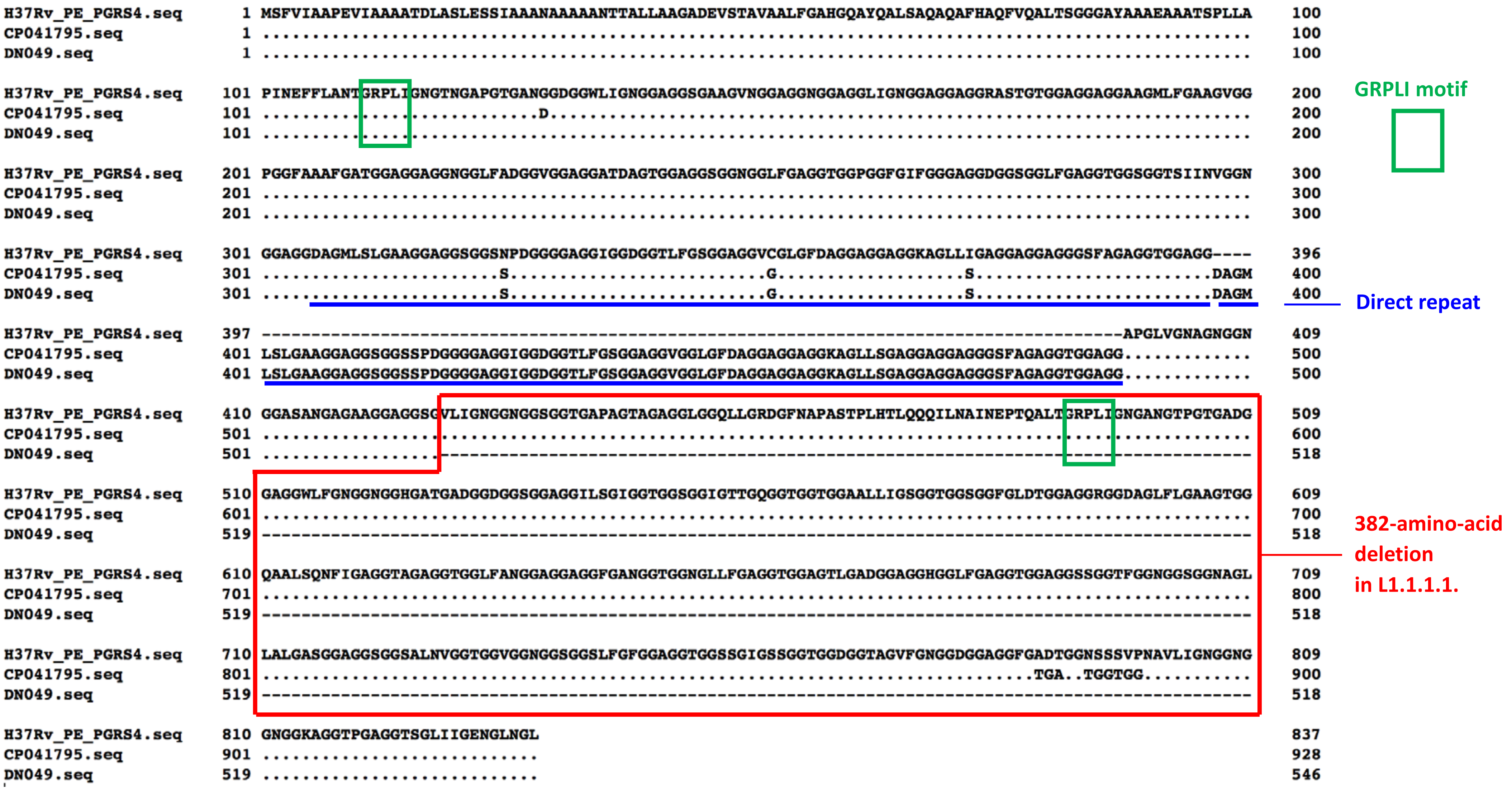

Supplementary Fig. S5: Comparison of RD900 region.

a) Nucleotide sequence alignment of the RD900 region. Nucleotide sequences from *pknH1* to *pknH2* of L1 Mtb DN-059 (Accession no. AP024455 in this study), Maf_GM041182 (L6 Mtb West African 2 or *Mycobacterium africanum* strain GM041182, NC_015758.1), and H37Rv (AL1234556.3) were aligned by the ClustalW module built into Genetyx-Mac ver.21 (GENETYX Corporation, Tokyo, Japan).

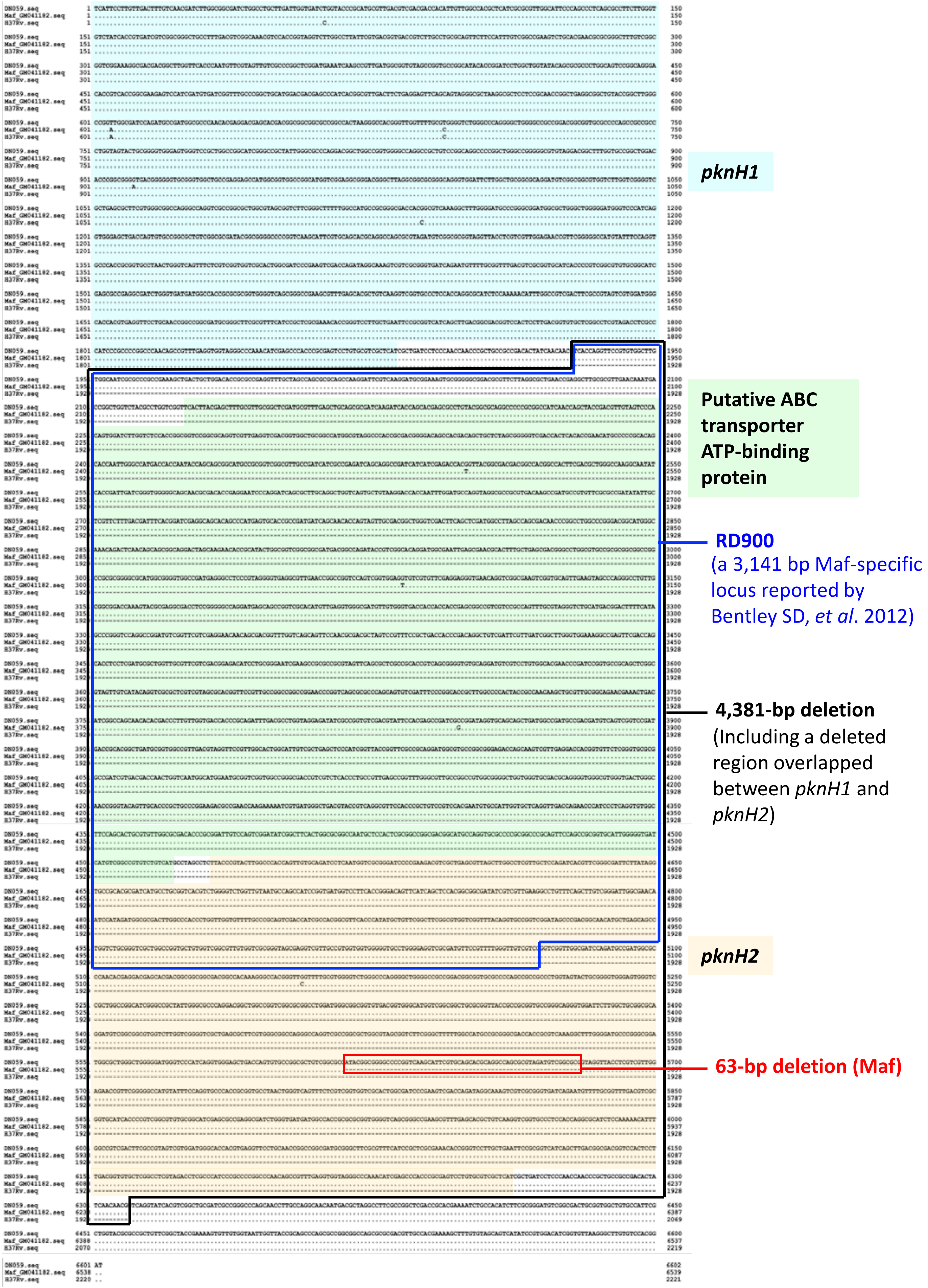

Supplementary Fig. S5b) and c) Alignment of deduced amino acid sequences of the putative ABC transporter ATP-binding protein (b) and PknH2 (c) for L1 Mtb DN-059 (Accession no. AP024455, this study), Maf_GM041182 (L6 Mtb West African 2, or *Mycobacterium africanum*, strain GM041182, NC_015758.1), *Mycobacterium tuberculosis* variant *bovis* (Mb3601, LR699570.1), and *Mycobacterium tuberculosis* variant *canettii* (CIPT 140010059, NC_015848.1) using the ClustalW module built into Genetyx-Mac ver.21 (GENETYX Corporation, Tokyo, Japan). bovis: *Mycobacterium tuberculosis* variant *bovis*, canettii: *Mycobacterium canettii*. Dots and dashes represent identical and deleted amino acids, respectively.

b)

```
DN-059.pep          1 MTDTADMITPNAPRLELRAAGRTWHAVAGREWSIGRASEADIRLDNPRVSRQHAVLEATPEGWVLVNLSTNGTFVDGQRVERLTVRQPITIFLGSASSGQ 100
Maf_GM041182.pep    1 .................................................................................................... 100
bovis.pep           1 .................................................................................................... 100
canettii.pep        1 .................................................................................................... 100

DN-059.pep        101 RVQLYPVAQSPTPTPASHPPAPPRPATPKPAQRQGETTVARPPTAFHAIDQLVVTIGRAPENTVVLNDLLVSRRHAILRRTGNRWELSDNASANGTYVNG 200
Maf_GM041182.pep  101 .................................................................................................... 200
bovis.pep         101 .................................................................................................... 200
canettii.pep      101 ................P.S................................................................................. 200

DN-059.pep        201 HRISRAVIGPTDIVGIGHQLLHLSGDRLVEYVDTGDISYQASNLRVVTNKGRVLLADVSFVLPQRSLLAVVGPSGAGKSTLLGALTGFRPAGNGTVRYDE 300
Maf_GM041182.pep  201 ........................A........................................................................... 300
bovis.pep         201 ........................A........................................................................... 300
canettii.pep      201 .................................................................................................... 300

DN-059.pep        301 RDLYDNYAELRHRIGFVPQDDILHTPLTVRRALNYAARLRFPQDVSVDERNQRIEEVLVELGLSTQADQRIDSLSGGQRKRTSVALELLTKPSLLFLDEP 400
Maf_GM041182.pep  301 .................................................................................................... 400
bovis.pep         301 .................................................................................................... 400
canettii.pep      301 .................................................................................................... 400

DN-059.pep        401 TSGLDPGYEKSVMQTLRKLADDGRSVVVVTHNIAHLNMCDRLLILAPGGRLAYFGPPQQALGYFNCTDFADLFTLLEHDTSTDWTGRFNASPLREALIGH 500
Maf_GM041182.pep  401 ...............................................................................N.................... 500
bovis.pep         401 .................................................................................................... 500
canettii.pep      401 .................................................................................................... 500

DN-059.pep        501 PAMRPARPAAARHARPVAQQSAFAQFAILCRRYLAVIAADRQYAVFLLVLPLLLSLFAHAVPGQAGLSLAKAIELKSTQPSQLLVLLIIGGALMGCAASI 600
Maf_GM041182.pep  501 .................................................................................................... 600
bovis.pep         501 .................................................................................................... 600
canettii.pep      501 .................................................................................................... 600

DN-059.pep        601 REIVKERAIYRREHGIGLSRGAYLASKLVVLTALTSLQALILGFLGVALLPPPDQSVILPWPSVEVAVAVVAVTVVSMMIGLLISAMIGNADRGMPLLVL 700
Maf_GM041182.pep  601 .................................................................................................... 700
bovis.pep         601 .................................................................................................... 700
canettii.pep      601 .................................................................................................... 700

DN-059.pep        701 VVMAQLVLCGGMFGVSGRPPLEQLSWLSPSRWAYAMAAATVDLNDLRRTAGGDQDPLWDYNVGSWLMAAGACAVQALVLVILIALQLKRIEPQRKARK   798
Maf_GM041182.pep  701 ..................................................................................................   798
bovis.pep         701 ..................................................................................................   798
canettii.pep      701 ..................................................................................................   798
```

c)

```
DN-059.pep            1 MSDAQDSRVGSMFGPYHLKRLLGRGGMGEVYEAEHTVKEWTVAVKLMTAEFSKDPVFRERMKREARIAGRLQEPHVVPIHDYGEVDGQMFLEMRLVEGTD   100
Maf_GM041182.pep      1 ....................................................................................................   100
bovis.pep             1 ....................................................................................................   100
canettii.pep          1 ....................................................................................................   100

DN-059.pep          101 LDSVLKRFGPLTPPRAVAIITQIASALDAAHADGVMHRDVKPQNILITRDDFAYLVDFGIASATTDEKLTQLGTAVGTWKYMAPERFSNDEVTYRADIYA   200
Maf_GM041182.pep    101 ..............................................................................................------   194
bovis.pep           101 ....................................................................................................   200
canettii.pep        101 ....................................................................................................   200

DN-059.pep          201 LACVLHECLTGAPPYRADSAGTLVSSHLMGPIPQPSAIRPGIPKAFDAVVARGMAKKPEDRYASAGDLALAAHEALSDPDQDHAADILRRSQESTLPGTA   300
Maf_GM041182.pep    195 ---------------.....................................................................................   279
bovis.pep           201 ....................................................................................................   300
canettii.pep        201 ....................................................................................................   300

DN-059.pep          301 AVTAQPPTMPTVTPPPIQAAPTGQPSWAPNSGPMPASGPTPTPQYYQGGGWGAPPSGGPSPWAQTPRKTNPWPFVAVAAAVVLVLVLGAIGIWIANRPDD   400
Maf_GM041182.pep    280 ....................................................................................................   379
bovis.pep           301 .........................................................................L..G.......................   400
canettii.pep        301 ...........................................................T........................................   400

DN-059.pep          401 NPKRNIATSPGTPTTTATTSLPATTTPTTAPASDPQTRLLSMLPSGYPTGTCKPTTPKPNSIWVNAVAMVDCGQNTNQGGPSRAIYGLFANPDKLKQAFN   500
Maf_GM041182.pep    380 ....................................................................................................   479
bovis.pep           401 ....................................................................................................   500
canettii.pep        401 ...................P................................................................................   500

DN-059.pep          501 DDIAAVELMNCPGEGPSPDGWHYNQTPDVTAGMIACGTYKNRPNVIWSNEAKLTLSDVFGDPATIEDLHNWWAKYG                           576
Maf_GM041182.pep    480 ............................................................................                           555
bovis.pep           501 ............................................................................                           576
canettii.pep        501 ............................................................................                           576
```

Supplementary Fig. S6: A 118-bp deletion in *furA* and ZERO-clade strains among the Da Nang (a) and southern Vietnam data sets (b), viewed by Integrative Genomics Viewer (IGV) version 2.3.88 (http://software.broadinstitute.org/software/igv/home).

a) **b)**

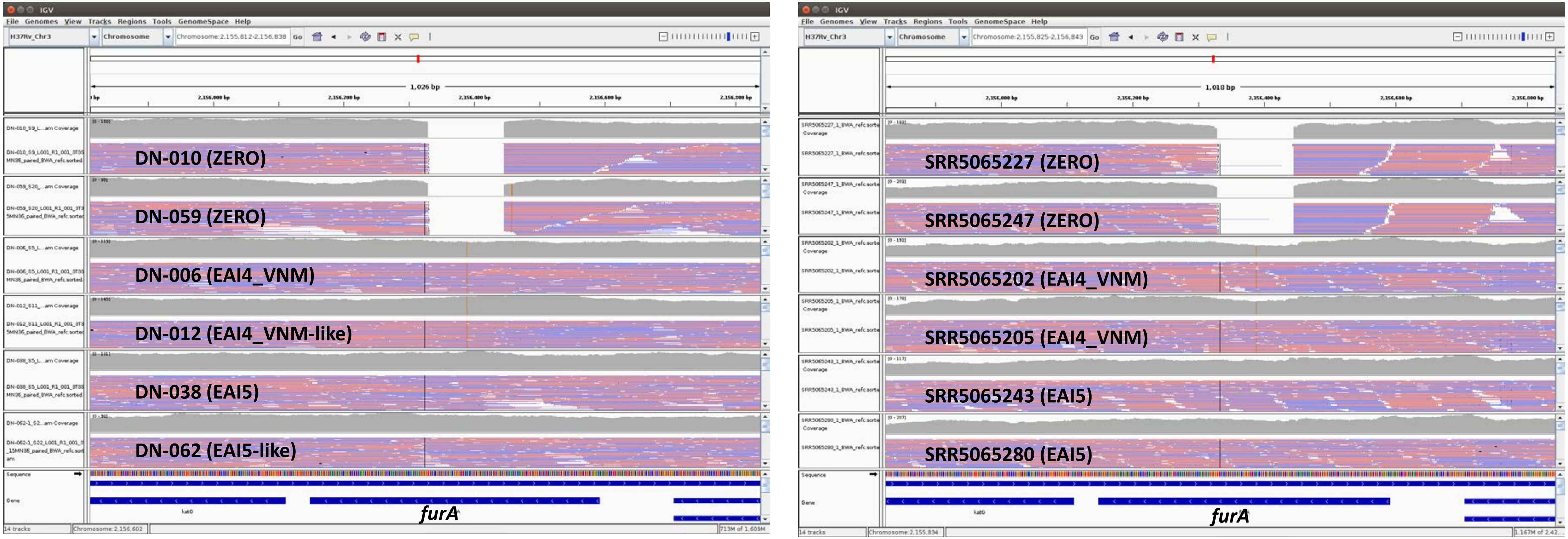

Supplementary Fig. S7: Genome assembly graphs of ZERO (a), EAI4_VNM (b), and Beijing (c) strains visualized with Bandage version 0.8.1 (https://github.com/rrwick/Bandage) and the distribution of IS*6110* copies detected with a BLAST search with the X17348 sequence as the query. Red triangles ▶: location of IS*6110* elements.

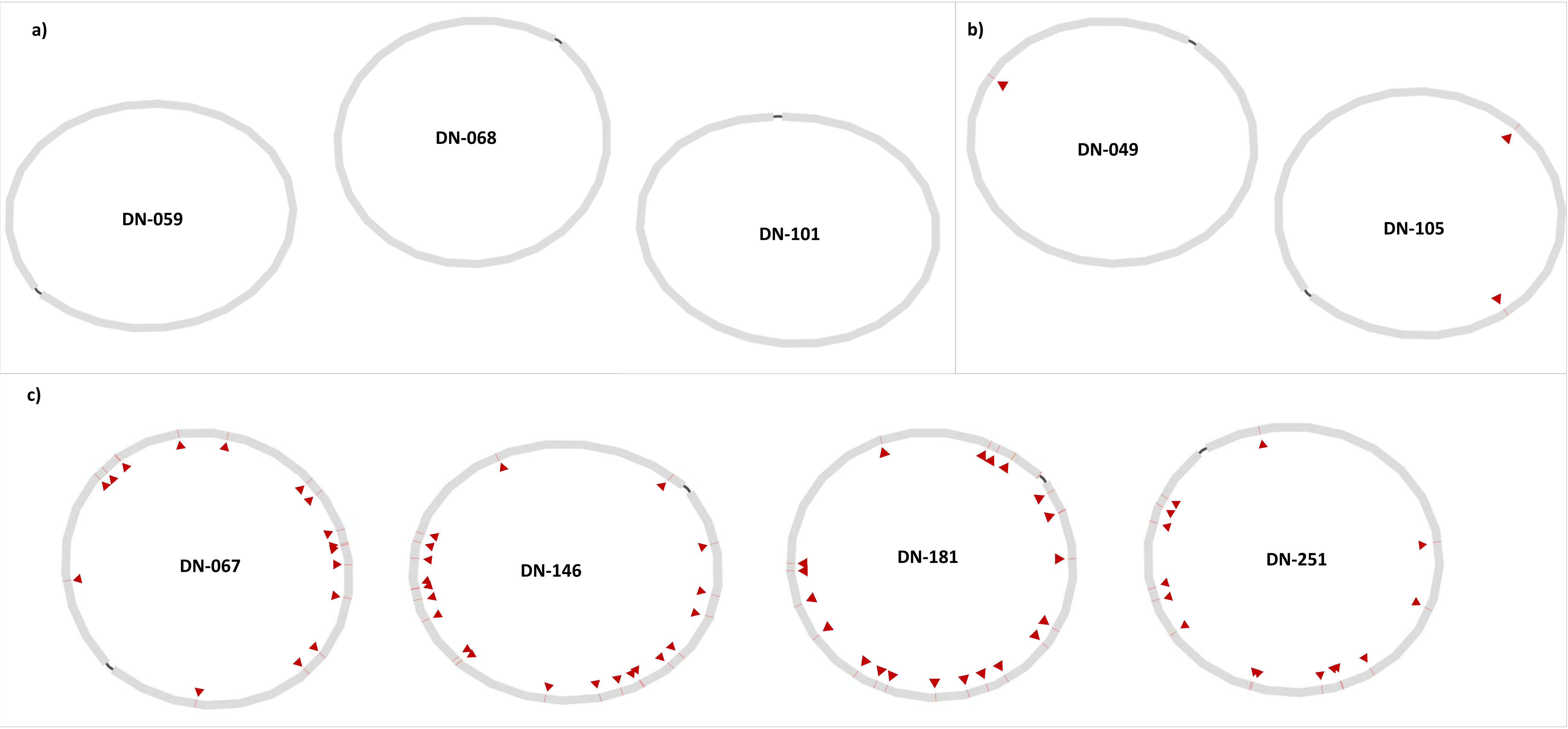

Supplement: Supplementary file 1 — Supplementary Information. [file 41598_2021_92984_MOESM1_ESM.pdf]
